# Single-cell analysis of bidirectional reprogramming between early embryonic states identify mechanisms of differential lineage plasticities in mice

**DOI:** 10.1016/j.devcel.2024.11.022

**Published:** 2024-12-26

**Authors:** Vidur Garg, Yang Yang, Sonja Nowotschin, Manu Setty, Eralda Salataj, Ying-Yi Kuo, Dylan Murphy, Roshan Sharma, Amy Jang, Alexander Polyzos, Dana Pe’er, Effie Apostolou, Anna-Katerina Hadjantonakis

**Affiliations:** 1 Developmental Biology Program, Sloan Kettering Institute, Memorial Sloan Kettering Cancer Center, New York, NY 10065, USA; 2 Biochemistry, Cell and Molecular Biology Program, Weill Cornell Graduate School of Medical Sciences, New York, NY 10021, USA; 3 Computational and Systems Biology Program, Sloan Kettering Institute, Memorial Sloan Kettering Cancer Center, New York, NY 10065, USA; 4 Joan & Sanford I. Weill Department of Medicine, Sandra and Edward Meyer Cancer Center, Weill Cornell Medicine, New York, NY 10021, USA; 5 Howard Hughes Medical Institute, New York, NY 10065, USA

**Keywords:** epiblast, primitive endoderm, ES cells, XEN cells, pluripotency, extra-embryonic endoderm, reprogramming, lineage plasticity, single-cell analysis, blastocyst

## Abstract

Two distinct lineages, pluripotent epiblast (EPI) and primitive (extra-embryonic) endoderm (PrE), arise from common inner cell mass (ICM) progenitors in mammalian embryos. To study how these sister identities are forged, we leveraged mouse embryonic (ES) and e*X*tra-embryonic *EN*doderm (XEN) stem cells – *in vitro* counterparts of the EPI and PrE. Bidirectional reprogramming between ES and XEN coupled with single-cell RNA and ATAC-seq analyses showed distinct rates, efficiencies and trajectories of state conversions, identifying drivers and roadblocks of reciprocal conversions. While GATA4-mediated ES-to-iXEN conversion was rapid and nearly deterministic, OCT4, KLF4 and SOX2-induced XEN-to-iPS reprogramming progressed with diminished efficiency and kinetics. A dominant PrE transcriptional program, safeguarded by GATA4, alongside elevated chromatin accessibility and reduced DNA methylation of the EPI underscored the differential plasticities of the two states. Mapping *in vitro* to embryo trajectories tracked reprogramming cells in either direction along EPI and PrE *in vivo* states without transitioning through the ICM.

## eTOC Blurb

Garg et al. use bi-directional reprogramming between embryonic (ES) and extra-embryonic endoderm (XEN) stem cells to probe transcriptional and epigenetic factors that direct lineage specification *in vivo*, and facilitate or prevent lineage inter-conversions. A strong endoderm transcriptional program, and distinct epigenetic state accompany extraembryonic endoderm specification and prevent lineage conversion.

## Graphical Abstract

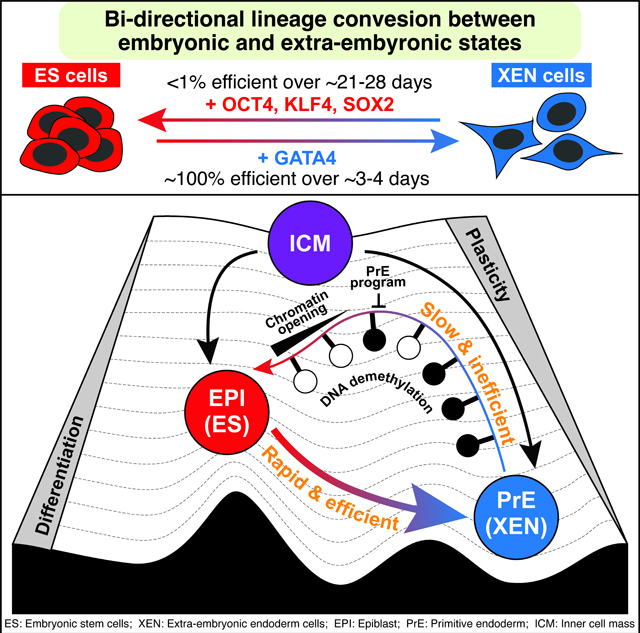

## INTRODUCTION

In mammals, the pluripotent epiblast (EPI), one of the first lineages specified during embryo development, emerges contemporaneously with its sister lineage, the extra-embryonic (primitive) endoderm (PrE), from a common progenitor population, the inner cell mass (ICM)^1,2^. The EPI and PrE lineages are distinct, with the EPI giving rise to the embryo-proper^3^, while the PrE will form the endoderm of the visceral and parietal yolk sacs, and part of the embryonic gut tube^4^. Though these lineages appear to be developmentally fixed, rare cells having committed to the PrE have been reported to switch to the EPI lineage and even vice versa^5–7^. Moreover, while PrE descendants contribute to the embryonic gut tube, they retain a partial transcriptional signature of their lineage of origin, suggesting a transcriptional, and perhaps epigenetic, memory^6,8^. These observations motivated us to seek a deeper understanding of how these two sister lineage identities are established and maintained.

A challenge in studying early mammalian embryos is their relatively small size, limited cell number, and availability. Stem cell models representing lineages of the embryo serve as *in vitro* alternatives, thus overcoming these limitations. Embryonic stem (ES) cells represent the pluripotent EPI^9,10^, and extra-embryonic endoderm stem (XEN) cells represent the PrE^11^ (Figure S1A). Like the preimplantation EPI, ES cells express naïve pluripotency-associated markers, such as *Nanog*, *Oct4*, and *Sox2*, while XEN cells are defined by expression of PrE markers *Gata6*, *Gata4*, *Sox17* and *Pdgfra*^12,13^. Upon reintroduction into the embryo, ES and XEN cells exclusively contribute cellular descendants to their lineage of origin^9–11^. Furthermore, stem cell models of blastocyst-derived lineages have been combined *in vitro* to form embryo-like structures that model various aspects of mammalian embryogenesis^14–19^.

Lineage conversion of *in vitro* stem cell models, along with cellular reprogramming to a pluripotent cell state, have been leveraged to examine the transcriptional and epigenetic mechanisms that control lineage identity, and determine the key milestones and bottlenecks in cell fate transition trajectories^13,20,21^. Transcription factor (TF) modulation can induce lineage conversions, emphasizing their importance in the specification and maintenance of lineage identity. Accordingly, endoderm-associated TFs GATA4 and GATA6 can convert ES cells to XEN cells (referred to as induced, or iXEN cells)^22–25^, which, along with observations made in mouse mutants^26,27^, have established them as core members of the XEN/PrE gene regulatory network. However, TF-induced reprogramming in the reverse direction – the conversion of XEN cells to ES/iPS cells – has not been demonstrated. Chemical reprogramming of somatic cells to induced pluripotent stem (iPS) cells reportedly transitions through a XEN-like state^28–30^, suggesting that XEN cells might represent a plastic state conferring the ability to acquire pluripotency. Alternatively, during TF-based reprogramming of somatic cells to iPS cells, emerging XEN-like cells have been described as a “dead-end”^31,32^, suggestive of a refractory cell state. Therefore, it remains unclear whether XEN cells can readily attain a pluripotent state.

Here, we demonstrate that ectopic expression of TFs (OCT4, KLF4 and SOX2) can reprogram XEN cells to a stable pluripotent state. However, by contrast to the reciprocal GATA4-mediated ES-to-iXEN conversion, which occurs over 4 days and is >95% efficient, XEN-to-iPS conversion requires ~3 weeks with an efficiency ~0.2%, suggesting a differential developmental plasticity of these two states. Leveraging this *in vitro* reciprocal lineage conversion system, bookended by EPI (ES/iPS) and PrE (XEN/iXEN) states, we charted the sequence of events that characterize these cell state transitions, to identify facilitators or barriers of lineage switching. We applied single-cell transcriptomic analyses (scRNA-seq) to establish a high-resolution map of EPI-PrE lineage conversions in both directions. These data outlined a linear, nearly deterministic, ES-to-iXEN conversion, and a heterogeneous and discontinuous XEN-to-iPS conversion with multiple intermediate terminal states. Dismantling of the XEN network by GATA4 knockout significantly increased reprogramming efficiency of XEN-to-iPS. Although direct comparison between the XEN-to-iPS and ES-to-iXEN trajectories indicated some differences at key transition points, comparison to *in vivo* scRNA-seq data from mouse embryos, showed that both *in vitro* trajectories largely tracked along *in vivo* trajectories during EPI and PrE lineage specification and maturation, but bypassed the ICM bipotent progenitor state. Bulk and single-cell assays for transposaseaccessible chromatin by sequencing (ATAC-seq) identified contrasting global levels and patterns of chromatin accessibility between ES/iPS and XEN/iXEN cells, and suggested that extensive and late chromatin opening during XEN-to-iPS conversion, partly due to the requirement for DNA demethylation, represents a major roadblock. Altogether, these observations highlight that EPI and PrE have drastically distinct cellular plasticities, and provide insights into the molecular basis of these differences.

## RESULTS

### OCT4, SOX2 and KLF4 can successfully reprogram XEN-to-iPS in a slow and inefficient manner

**Figure 1.**
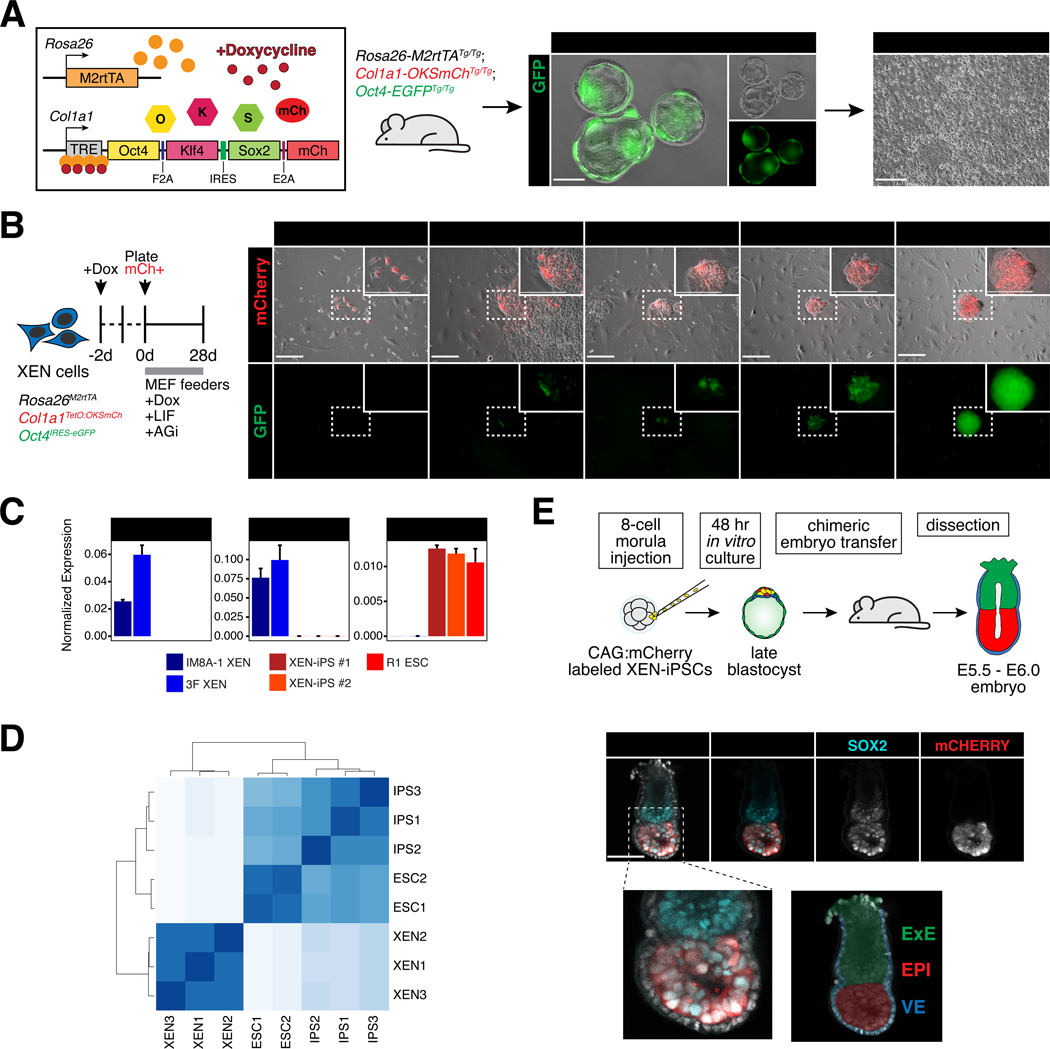
*Oct4*, *Sox2* and *Klf4* can successfully reprogram XEN cells in a slow and inefficient manner. **(A)** XEN cells were derived from mice homozygous for 3 alleles (*R26:M2rtTA*^*Tg/Tg*^; *Col1a1:OKSmCh*^*Tg/Tg*^; *Oct4:EGFP*^*Tg/Tg*^ referred to as ‘3F’). **(B)** *(Left)* Experimental scheme describing the reprogramming conditions used for XEN cells. *(Right)* Brightfield and fluorescent images of XEN cells during the reprogramming time course. Scale bars represent 250μm. **(C)** RT-qPCR data for several XEN and ES markers in wild-type XEN (IM8A-1), 3F XEN, two XEN-iPS lines, and wild-type ES cells (R1). Individual bars show mean expression of three technical replicates normalized to mean expression of two reference genes: *Actb* and *Gapdh*; error bars represent standard deviation. **(D)** Unsupervised hierarchical clustering of bulk RNA-seq data from XEN-iPS, wild-type ES, and XEN cells. **(E)** *(Top)* Experimental scheme outlining the generation of XEN-iPS chimeric embryos. *(Bottom)* Maximum intensity projection of 5 optical sections from a confocal image of a chimeric embryo stained with indicated markers. Nuclei labeled with DAPI, mCherry counter stained with anti-RFP. Scale bars represent 100μm.

To test the potential for XEN cells, representing the PrE, to reprogram to iPS cells, representing the epiblast, we derived XEN cells from a transgenic doxycycline-inducible “reprogrammable” mouse strain used in previous studies for reprogramming several somatic cell types, including mouse embryonic fibroblasts (MEFs) and hematopoietic lineages (Figure 1A)^33–35^. Doxycycline (‘dox’) induction with a constitutively expressed reverse tetracycline transactivator (Rosa26-M2rtTA) drives the expression of a polycistronic cassette consisting of *Oct4*, *Klf4*, *Sox2*, and an *mCherry* reporter (referred to as 3-factor, or ‘3F’). An additional *Pou5f1*-IRES-EGFP reporter allele enables detection of endogenous *Oct4* activation (*Oct4*-GFP), silent in XEN cells^11,36^. Blastocysts harboring all three alleles were used to derive multiple XEN cell lines using standard protocols^11^.

Embryo-derived 3F XEN cells were treated with dox for 2 days prior to sorting the mCherry+ fraction (~15–30% of total population) to ensure selection of cells expressing the reprogramming cassette. Sorted mCherry+ cells were plated for reprogramming in serum/LIF medium with ascorbic acid and GSK3βi (‘AGi’), an enhanced reprogramming protocol which improves reprogramming efficiency in somatic cells, including conditions without exogenous *Myc* expression as in our experiments^33^ (Figure 1B). Under these conditions, we could derive transgene-independent, iPS-like, *Oct4*-GFP+ colonies, although at very low frequency (<1%) and slow kinetics (~18–24 days). Derived XEN-iPS cells had silenced XEN/PrE markers, including *Gata6*, *Gata4* and *Pdgfra*, and upregulated pluripotency-associated markers, including *Nanog*, *Esrrb*, *Fgf4*, *Zfp42* (*Rex1*), *Dppa3* (*Stella*) and *Utf1*, at levels similar to wild-type ES cells^37^ (Figure 1C and S1B-C). Hierarchical clustering and principal component analysis of bulk RNA-seq data clustered XEN-iPS cells together with ES cells, and apart from parental or wild-type XEN lines (Figure 1D and S1D). To further assess developmental potential and lineage restriction of XEN-iPS cells, we generated chimeric embryos by injecting cells into 8-cell (morula) stage host embryos, prior to ICM lineage specification. A constitutively expressed CAG:mCherry construct was introduced into XEN-iPS cells to readily identify their descendants in embryo chimeras (Figure 1E, top). Unlike their parental XEN cells^11^, XEN-iPS contributed exclusively to the epiblast compartment and were excluded from PrE-derived visceral and parietal endoderm tissues (Figure 1E and S1E). These results demonstrate that TF-induced reprogramming of XEN cells can generate *bona fide* iPS cells, albeit in a slow and inefficient manner.

### Bidirectional reprogramming of XEN and ES cells show drastically different kinetics and efficiencies of conversion

**Figure 2.**
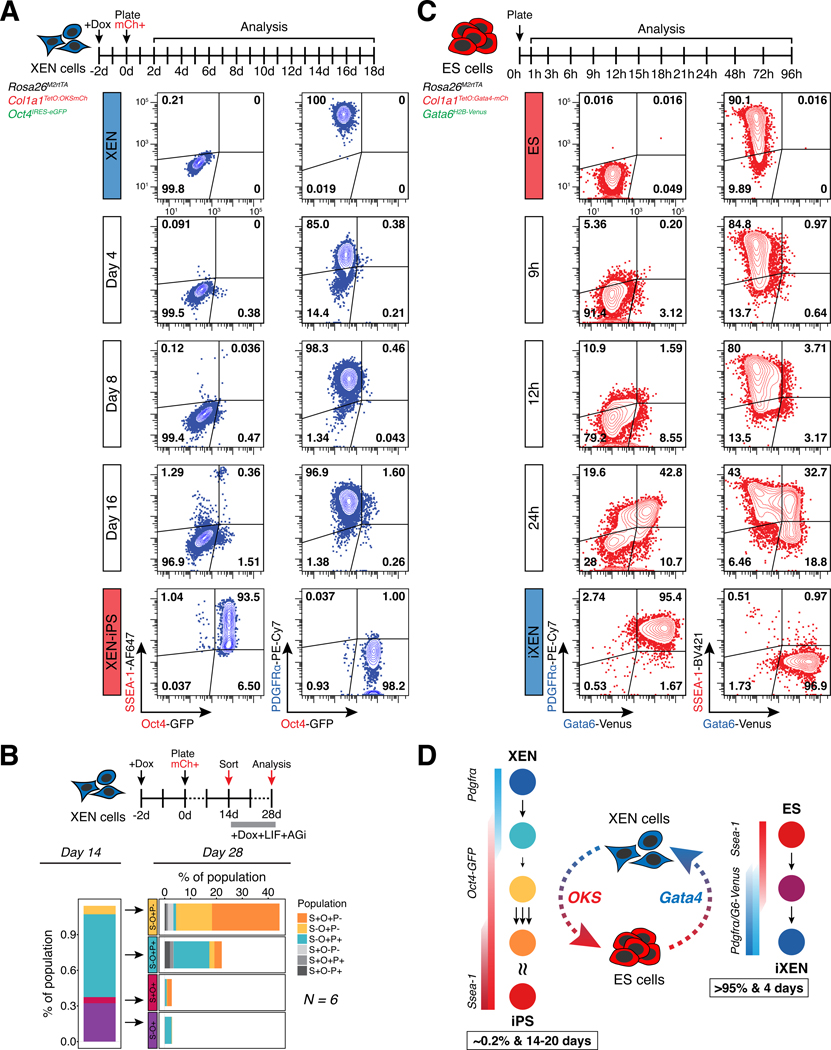
Reciprocal lineage conversions of XEN and ES cells have drastically different kinetics and efficiencies of conversion. **(A)** Time course flow cytometry-based analysis of pluripotency-associated markers SSEA-1 and *Oct4*-GFP, and XEN-associated marker PDGFRα during XEN reprogramming. Representative contour plots show expression of SSEA-1 (AlexaFluor647-conjugated), *Oct4*-GFP and PDGFRα (PE-Cy7-conjugated) at days 2, 8 and 16 of reprogramming. Population percentage indicated within each gate. **(B)** *(Top)* Tracking the reprogramming efficiencies of four major subpopulations sorted at day 14 of reprogramming. At day 28, populations arising from each sorted subpopulation were determined using flow cytometry. *(Bottom, left)* Stacked bar charts depicting the mean proportion of the population represented by each subpopulation at day 14, and at day 28 *(bottom, right)*. S-O-P+ subpopulation constitutes the remainder of the population (not shown) to amount to 100% and represents non-reprogramming XEN cells. N = 6 (2 independent experiments with 3 replicates each). **(C)** Time course tracking of ES-to-iXEN conversion using flow cytometry analysis of SSEA-1, PDGFRα and *Gata6*-Venus reporter. Representative contour plots show expression of PDGFRα (PE-Cy7-conjugated), *Gata6*-Venus and SSEA-1 (BV421-conjugated) at indicated timepoints. Population percentage is indicated within each gate. **(D)** Summarized schematic of XEN-to-iPS and ES-to-iXEN lineage conversions. *(Left)* Hypothesized reprogramming route taken by XEN cells. Sizes and number of arrows reflect the likelihood of cells progressing from one state to the next (smaller/single arrows = few cells progress; larger/more arrows = many cells progress). Reprogramming XEN cells initiate expression of *Oct4*-GFP, then downregulate PDGFRα. This downregulation step represents a bottleneck during reprogramming since few *Oct4*-GFP+ cells progress to this state. Following PDGFRα downregulation, a large proportion of cells upregulate SSEA-1. *(Right)* Hypothesized lineage conversion of ES to iXEN cells. Initial upregulation of both, PDGFRα and *Gata6*-Venus, is followed by downregulation of SSEA-1. No obvious bottlenecks are detected during ES-to-iXEN conversion.

To investigate the trajectory of XEN reprogramming toward iPS cells, and identify intermediate populations and rate limiting steps, we used flow cytometry to track the activation dynamics of two pluripotency-associated markers – the *Oct4*-GFP reporter and surface marker SSEA-1^36,38,39^ – as well as silencing of the XEN marker PDGFRα^40–42^ (Figure 2A and S2A). We consistently observed asynchronous and independent activation of *Oct4*-GFP (O+) and SSEA-1 (S+), with a small group of cells (~0.5%) only expressing *Oct4*-GFP (O+) as early as day 4 of reprogramming, while another subset (~0.1%) only expressed SSEA-1 at later stages (~day 8). Cells co-expressing both pluripotency markers, SSEA-1 and *Oct4*-GFP (S+O+), were detected between days 14–16 of reprogramming. By contrast, PDGFRα (P+) expression persisted in the majority of cells even after upregulation of *Oct4* and/or SSEA-1, and was downregulated only in late stages of reprogramming, at ~day 16. To determine whether up- or downregulation of these markers represented more advanced or delayed intermediate states, we sorted four different subpopulations at day 14 (S+O-P+, S+O+P+, S-O+P+ and S-O+P-) and re-plated them in reprogramming conditions (serum/LIF+AGi+dox) for 14 additional days prior to analysis (Figure 2B). Cells expressing SSEA-1 alone, or with, *Oct4*-GFP (S+O- or S+O+) early during reprogramming did not contribute significantly to the final S+O+P- iPS-like population (<5%), and often regressed to a S-state, suggesting that SSEA-1 is not a predictive marker of XEN reprogramming. Cells expressing *Oct4*-GFP while silencing PDGFRα (S-O+P-) were the only intermediate population with advanced reprogramming potential (>20%). These results document the slow and heterogeneous nature of XEN-to-iPS reprogramming, and suggest that silencing of the XEN program is a critical rate-limiting step.

Although the reprogramming efficiency and kinetics varied among different 3F XEN lines, they remained consistently low (~0.2%) and slow (~20 days) across all lines tested (Figure S2B). In contrast, the opposite ES-to-iXEN fate transition has been reported to be efficient^24,25,43^. To directly compare reciprocal interconversion efficiencies and trajectories, we used an ES cell line engineered to express *Gata4*-mCherry fusion protein upon dox-induction^24^. This system enables rapid and efficient conversion to a XEN-like state (induced XEN or iXEN) over the course of 4 days, with >95% of cells silencing pluripotency markers (e.g. SSEA-1), and activating XEN markers, including PDGFRα and endogenous *Gata6* linked to a H2B-Venus reporter (*Gata6*-Venus)^24,44^ (Figure 2C and S2A). Although activation of XEN-associated PDGFRα and *Gata6*-Venus occurs as early as ~9–12h, most cells continue co-expressing SSEA-1, which is subsequently silenced.

Together, these results document prominent differences in the efficiency and kinetics of ES and XEN cell interconversions (Figure 2D). Transitioning from XEN-to-iPS states is slow and involves a heterogeneous trajectory, in contrast to the opposite ES-to-iXEN conversion. This argues that XEN cells are “locked” into a less plastic state with a stable transcriptional program resistant to reprogramming and silencing, as documented by the persistent expression of XEN markers until very late stages of conversion. In further agreement, nascent iXEN cells (PDGFRα+) derived from iPS cells using transient *Gata4* expression (for 5 or 7 days) lose their ability to re-acquire an iPS state upon dox-inducible OKS expression – with reprogramming efficiencies similar to embryo-derived XEN cells (Figure S2C-D). Notably, despite their distinct kinetics and efficiencies, conversions in both directions (ES-to-iXEN and XEN-to-iPS) appear to transition through a state where both ES and XEN markers are co-expressed, suggesting an, at least partially, overlapping trajectory.

### Single-cell transcriptomics identifies a discontinuous XEN-to-iPS reprogramming trajectory with multiple end-states in contrast with the linear ES-to-iXEN conversion

**Figure 3.**
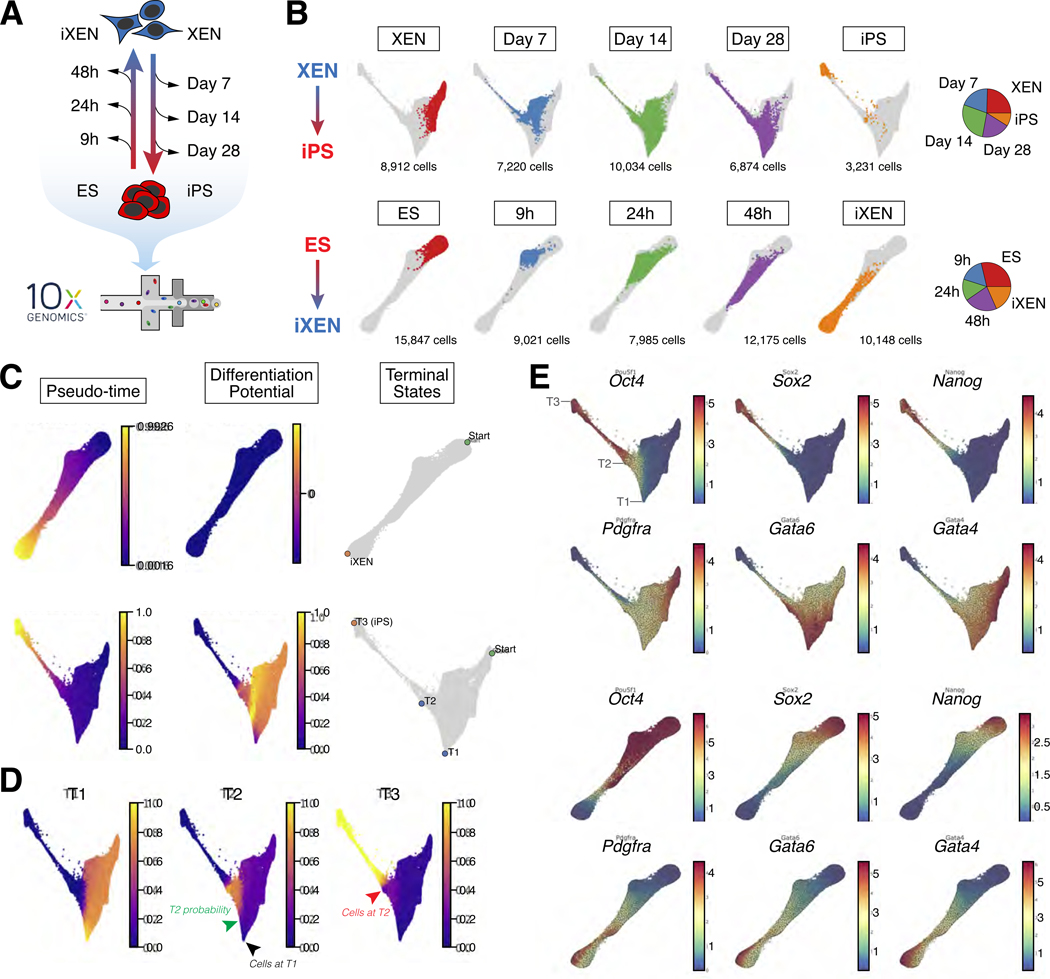
scRNA-seq analyses of XEN-to-iPS and ES-to-iXEN conversions. **(A)** Experimental scheme outlining cells and lineage conversion timepoints assayed by scRNA-seq. **(B)** Force-directed layouts showing XEN-to-iPS *(top)* and ES-to-iXEN *(bottom)* conversion trajectories. Individual plots highlight individual timepoints; pie charts indicate the proportion of the trajectory represented by each timepoint. **(C)** Palantir determined pseudotime ordering, differentiation potential, and terminal states of ES-to-iXEN *(top)* and XEN-to-iPS *(bottom)* trajectories. **(D)** Branch probabilities of terminal states determined by Palantir in the XEN-to-iPS trajectory. Black arrowhead indicates cells at T1 with low probability of differentiating to T2. Green arrowhead indicates where T2 probability increases. Red arrowhead indicates cells at T2, having a non-zero probability of acquiring the T3 state. **(E)** Gene expression of XEN and pluripotency-associated markers. Each cell is colored by its MAGIC imputed expression level. T1, T2 and T3 terminal states indicated for the XEN-to-iPS trajectory.

To map the trajectories of these bidirectional cell fate transitions at a single-cell level, we performed scRNA-seq at five distinct timepoints during each conversion. Specifically, we profiled embryo-derived XEN or ES cells and fully reprogrammed iPS or iXEN cells (representing starting and end states), as well as cells from early, middle and late stages of reprogramming in each direction based on marker expression (Figure 3A). Samples were collected in technical duplicates, with ~8,000 cells sampled from each replicate and timepoint (i.e., ~16,000 cells total per timepoint; ~160,000 cells total across all timepoints and both trajectories). Since XEN-to-iPS reprogramming is an inefficient process, to avoid potential underrepresentation of rare cells undergoing successful reprogramming, we sorted four intermediate subpopulations based on SSEA1 and *Oct4*-GFP expression (see Figure 2B and S2A) – S-O-, S+O-, S+O+ and S-O+ – and reconstituted them in roughly equal proportions prior to droplet encapsulation (Figure S2E). We followed this approach for all three intermediate timepoints for the XEN-to-iPS trajectory, representing day 7, day 14 and day 28 of reprogramming. A similar strategy was used for the early (9h) timepoint for the ES-to-iXEN trajectory (Figure S2E).

To gain insights into when and how lineage conversions occur, we combined data from all replicates and timepoints separately for each reprogramming trajectory and projected them on a Force Directed Layout (FDL) (Figure 3B). We then applied Palantir^45^, to automatically identify the terminal states, and the branch probabilities for each cell reaching each of the identified states. The differentiation potential (entropy) of these branch probabilities represents the uncertainty of future cell fate (Figure 3C and 3D). Consistent with the rapid and efficient dynamics of ES-to-iXEN conversion, we identified a linear trajectory and a singular terminal state that cells eventually reach (Figure 3B and 3C). By contrast, during XEN reprogramming we detected three distinct terminal states, ‘T1’, ‘T2’ and ‘T3’, highlighting the inefficient and more heterogeneous nature of XEN-to-iPS conversion. The most prominent T1 state represented a XEN-like state with high expression of XEN genes, absence of pluripotency gene expression, and upregulation of AP-1 family members (such as *Jun, Fos, Atf3*), which have been shown to inhibit somatic cell reprogramming in different contexts^46–48^(Figure 3E and S2F; Table S1). On the other hand, T3 reflected the iPS/EPI state characterized by expression of pluripotency-associated genes and silencing of the XEN/PrE program. Finally, the T2 state was defined by expression of multiple XEN genes along with some pluripotency-associated markers, such as *Oct4* (endogenous), and *Rex1*^49–51^, in agreement with our flow cytometry data showing transient co-expression of the EPI and PrE markers during reprogramming.

Palantir inferred that cells at the start of the trajectory had a high probability of reaching T1 (Figure 3D). However, cells at T1 showed negligible probability of acquiring T2 or T3 states, indicating that T1 might serve as a ‘sink/dead-end’ during XEN-to-iPS reprogramming (Figure 3D, black arrowhead). These results suggest that the inefficiency of reprogramming may in part be due to cells being diverted toward a stable refractory T1 state. However, cells at T2, showed a low but non-zero probability of reaching the T3 state (red arrowhead), serving as a potential bottleneck toward successful reprogramming, which may represent an intermediate more plastic state than T1. Once cells passed the T2 state, the probability of reaching T3, the end-point reprogrammed state, sharply increased. This phase of the trajectory was characterized by downregulation of XEN-associated genes accompanied by activation of additional pluripotency-related genes (Figure 3E and S2G). Notably, *Oct4* (as well as other pluripotency regulators, e.g., *Rex1* and *Klf9*) and *Dnmt3l* were upregulated prior to the T2 state, and their activation coincided with increased probability of cells reaching T2 (Figure 3D and 3E, green arrowhead), suggesting this to be an important but insufficient step for XEN-to-iPS reprogramming in agreement with our cell sorting analyses (see Figure 2B). Accordingly, *Oct4*-GFP and PDGFRα co-expressing cells (O+P+) generated from transient expression of *Gata4* in iPS cells showed a greater propensity for reprogramming than nascent iXEN (O-P+ cells), or embryo-derived XEN cells (Figure S2C-D), suggesting that *Oct4*-expressing T2-like cells are more amenable to reprogramming than starting *Oct4*-negative XEN cells.

In sum, our scRNA-seq data support XEN-to-iPS reprogramming as a heterogeneous process with multiple terminal states representing major roadblocks that must be bypassed to silence the PrE program and establish the EPI state.

### XEN and ES reprogramming approximate *in vivo* cell states, but not ICM progenitor cells

**Figure 4.**
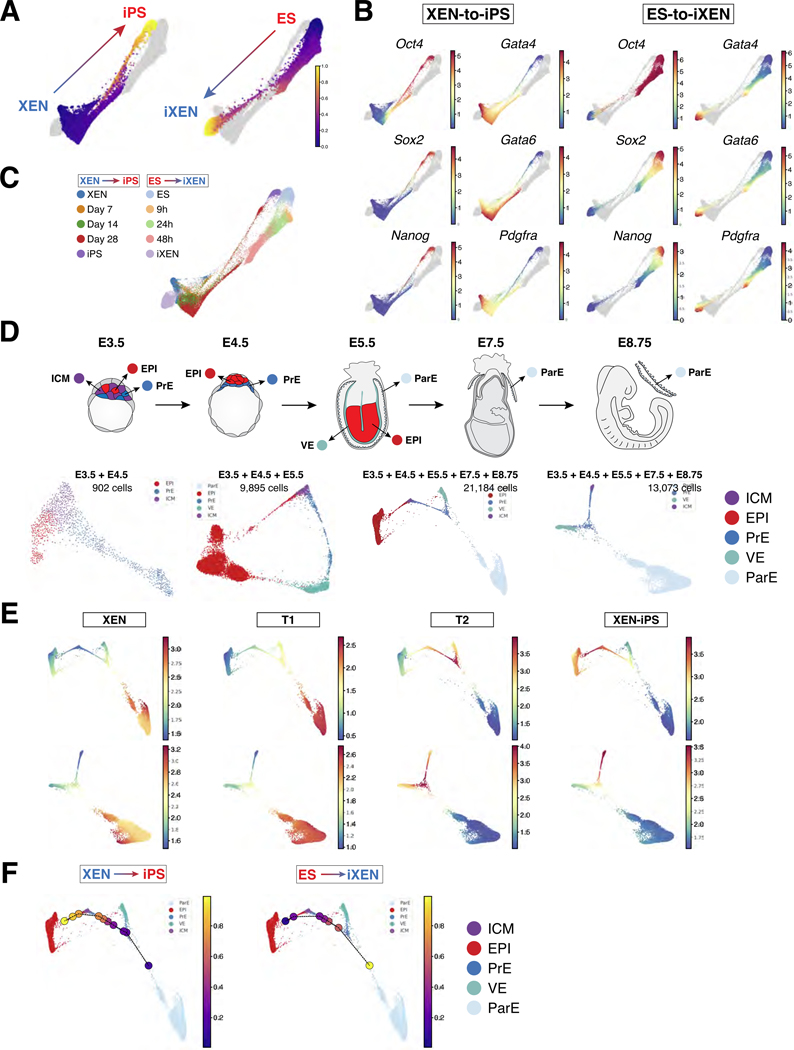
XEN-to-iPS and ES-to-iXEN conversion trajectories approximate *in vivo* cell states. **(A)** Force-directed layout of combined XEN-to-iPS and ES-to-iXEN trajectories based on Harmony^6^ integration (number of cells = 91,447). XEN-to-iPS *(left)* or ES-to-iXEN *(right)* are highlighted. Cells are colored by Palantir pseudotime, computed separately as in Figure 3. **(B)** Gene expression of pluripotency and XEN-associated markers displayed in the combined trajectory. Each cell is colored by its MAGIC imputed expression level in the individual XEN-to-iPS or ES-to-iXEN trajectories, as labeled. **(C)** Force-directed layout of combined trajectories (as in panel A) with individual timepoints from each trajectory colored as indicated. **(D)** *(Top) in vivo* embryo stages and tissues (labeled) profiled by scRNA-seq. *(Bottom)* Force-directed layouts of combined *in vivo* stages and lineages as labeled above, and color-coded as indicated by cell type. **(E)** Gene expression signatures of XEN, XEN-iPS, and T1 and T2 terminals states mapped onto force-directed layouts of combined *in vivo* states including *(top)* or excluding *(bottom)* the EPI lineage. **(F)** Visualization of *in vitro* XEN-to-iPS *(left)* or ES-to-iXEN *(right)* bins mapped onto the combined *in vivo* trajectory. Individual circles represent single *in vitro* bins and are color coded according to pseudotime.

The drastically different efficiencies of the two lineage interconversions might suggest that they transition through unique intermediate states. To examine this possibility, we used Harmony^6^ to combine all transcriptomes from XEN-to-iPS and ES-to-iXEN trajectories (Figure 4A-C). Merging of all datasets indicated that while the starting and end states of the two trajectories (ES/iPS and XEN/iXEN) are similar, the trajectories show differences during the transition stages of lineage conversion. To quantify this difference, we first utilized the individual trajectories to bin cells along the XEN-to-iPS and ES-to-iXEN conversions, respectively (Figure S3A-B). We then computed the average phenotypic distance between each pair of bins and visualized the pairwise distance matrix as a heatmap (Figure S4A), which illustrated similarity at the terminal points of the transition and dissimilarity at intermediate stages. To further understand this dissimilarity, we computed the differentially expressed genes at intermediate states during each conversion. For this, we first aligned the two trajectories using another Harmony algorithm^52^, and clustered the cells at intermediate stages, followed by differential expression analysis using MAST^53^ (Figure S4B-C; Table S2). Genes upregulated in the ES-to-iXEN trajectory included chromatin modifiers, such as *Jarid2*, *Hmga2* and *Arid1a*, while genes expressed during XEN-to-iPS conversion showed enrichment of mitochondrial and cellular metabolism-related genes. These findings indicated that reprogramming between EPI and PrE lineages involves transitioning through divergent cellular states, likely driven by unique epigenetic and metabolic regulators associated with starting XEN and ES states^54–58^. Additionally, transition states during XEN-to-iPS reprogramming upregulated genes encoding several members of the AP-1 complex, such as *Atf3*, *Fos*, *Jun*, *Junb*, likely reflecting the “refractory” T1 state that cells acquired during conversion. We confirmed these findings with an alternative batch alignment method using Harmony^6^ and Spectral Clustering for grouping cells (see Methods for details).

We next sought to determine the degree to which *in vitro* reprogramming trajectories resembled cell states *in vivo* during the emergence and differentiation of EPI and PrE lineages. For this, we leveraged our published scRNA-seq datasets from early (E3.5 and E4.5) mouse embryos^6^ to compile a reference subset of *in vivo* cells comprising uncommitted ICM progenitors, their derivative EPI and PrE cell lineages (Figure 4D and S4D), as well as the post-implantation EPI and visceral endoderm (VE) at E5.5. Since XEN cells have been suggested to resemble the parietal endoderm (ParE) branch of the PrE lineage due to their morphology, marker expression and lineage contribution in embryo chimeras^11,59–61^, we re-analyzed and appended our published scRNA-seq data of ParE cells collected from E7.5 and E8.5 embryo parietal yolk sacs^6^.

We first used Harmony^6^ to aggregate the *in vivo* timepoints, then used the combined *in vivo* trajectories, with or without the EPI lineage (to highlight the endoderm lineage), to identify the nearest *in vivo* states resembling the starting and terminal states (T1, T2 and T3/iPS) of XEN-to-iPS conversion (Figure 4D and S4D). We visualized the average expression of genes significantly differentially expressed (Figure S2F, see Methods) in each of the terminal states (Figure 4E). The starting XEN state mapped predominantly to ParE in the combined *in vivo* trajectory, providing an unbiased transcriptional basis for its ParE-like character^11^. The terminal T1 state also mapped to ParE, supporting the notion that T1 represents a stable state refractory to successful reprogramming (see Figure 3C-D). In contrast, T2 cells showed closer proximity to PrE indicative of their progression away from the starting ParE state during reprogramming. This was also consistent with co-expression of some pluripotency-associated markers, observed in our flow cytometry and scRNA-seq datasets, and in the PrE, and recently described extra-embryonic endoderm stem cells resembling the blastocyst PrE^62–65^. Finally, T3, or XEN-iPS, cells mapped to the EPI as expected for pluripotent cells.

We next mapped the *in vitro* XEN-to-iPS and ES-to-iXEN trajectories on the *in vivo* datasets to determine their progression along *in vivo* developmental states. We used Harmony^6^ to combine all *in vivo* and *in vitro* cell transcriptomes to derive a common augmented representation. The *in vivo* and *in vitro* trajectories were separately divided into equal-sized pseudotime bins and the closest *in vivo* bin for each *in vitro* bin was identified in the augmented representation (Figure S3 and S4E-F). In agreement with the previous analysis, we noted that the similarity to ParE persisted from starting XEN to T1 states. As cells progressed to the T2 state, they approximated *in vivo* PrE, particularly at E4.5, progressing to E3.5 PrE, before moving to EPI at E3.5 and, eventually, E4.5 EPI (Figure 4F and S4E). Cells undergoing ES-to-iXEN conversion followed a similar but opposite path along the *in vivo* trajectory, though not identical, reflecting differences noted previously, but suggesting that both conversions bore resemblance to cell states present *in vivo* (Figure 4F and S4F). Notably, neither XEN-to-iPS nor ES-to-iXEN trajectories transitioned through a state that resembled uncommitted ICM progenitors. These observations were consistent irrespective of the size and number of bins used, and given the high resolution of our scRNA-seq data, showed that EPI and PrE interconversions *in vitro* do not transition through an ICM-like state. Moreover, our observed toggling of cells between the EPI and PrE branches supports a model of bistability over tristability of EPI, PrE and ICM states, as has been suggested in studies modeling ICM development^24,26,66,67^.

### Silencing of the XEN program is a bottleneck for XEN-to-iPS reprogramming

**Figure 5.**
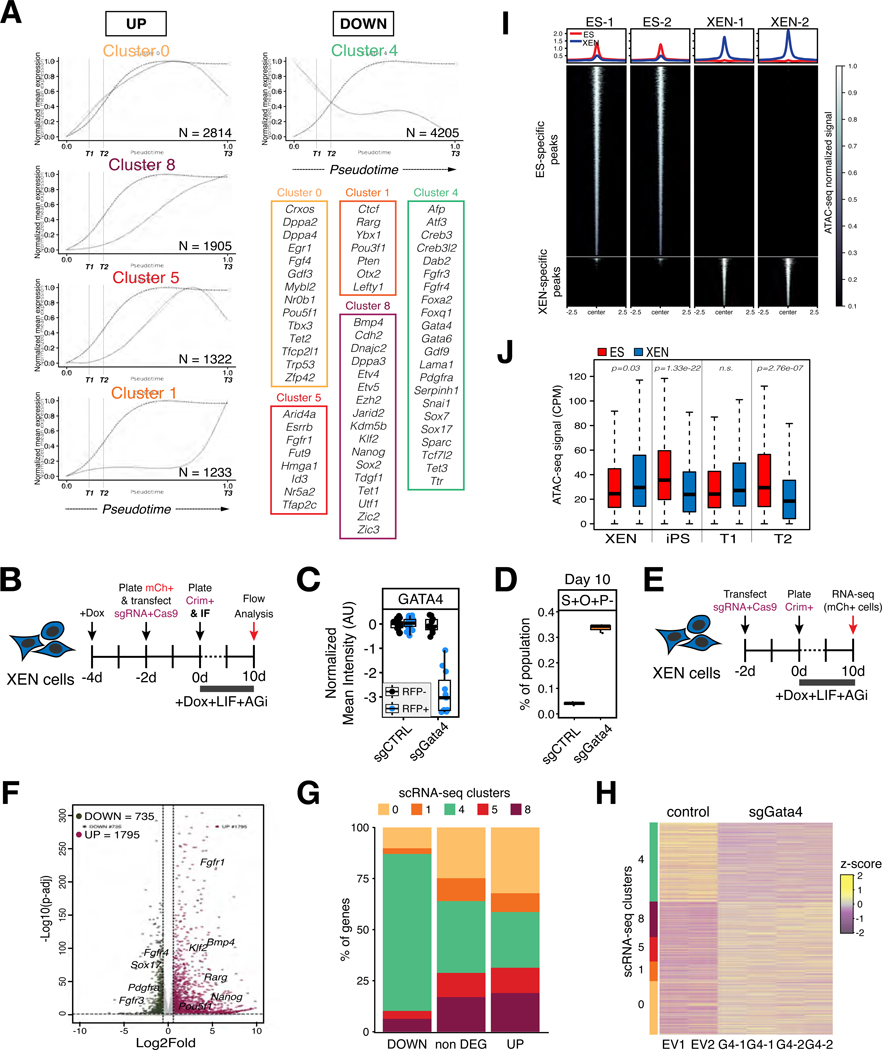
XEN transcriptional network presents a roadblock for XEN-to-iPS reprogramming. **(A)** Gene expression trends over pseudotime for XEN-to-iPS reprogramming. Plots show mean expression trend of all genes within each cluster. Dotted curve indicates probability of acquiring the T3/iPS state. Vertical dotted lines indicate T1 and T2 terminal states along the pseudotime axis. Representative genes for each cluster are highlighted in boxes. N = number of genes in each cluster. **(B)** Reprogramming efficiency following *Gata4* perturbation. XEN cells were transfected with plasmids expressing Cas9 and sgRNAs targeting *Gata4* or control. Immunofluorescence staining determined successful knockout of GATA4 expression (panel C). Following 10 days of reprogramming, resulting percentage of iPS-like cells was determined using flow cytometry (panel D). **(C)** Box plots showing relative reduction in anti-GATA4 fluorescence immunostaining in transfected XEN cells. Individual points represent relative fluorescence intensity in individual cells as arbitrary units and normalized to untransfected (RFP-) cells within the same well (see images in supplementary Figure S5C). **(D)** Box plots showing proportion of iPS-like cells (S+O+P-) in the entire population at day 10 of reprogramming following GATAT4 KO. N = 3. **(E)** Experimental timeline of GATA4 KO and sample collection for RNA-seq 10 days after the start of reprogramming. **(F)** Volcano plot showing significantly up/downregulated genes in cells transfected with *Gata4-*targeting sgRNA compared with control. **(G)** Stacked bar chart depicting the percentage of downregulated, upregulated, and non-differentially expressed genes that belong to the gene expression trend clusters shown in (A). **(H)** Heatmap representation of gene expression changes (z-score) between empty vector and *Gata4* sgRNA transfected cells (two different sgRNAs were used, G4–1 and G4–2), and association with the clusters shown in (A). **(I)** Tornado plots of normalized ATAC-seq signal in XEN and ES cells around cell type-specific peaks. ATAC-seq signals are shown for 2.5kb up/downstream of peak centers. **(J)** Box plots showing relative chromatin accessibility (normalized ATAC-seq signal) in XEN and ES cells of genes enriched in XEN, XEN-iPS, T1 and T2 terminal states.

Noting the discontinuous and inefficient nature of XEN-to-iPS reprogramming, we sought to further explore the transcriptional determinants and roadblocks of this lineage conversion. We first tracked gene expression changes along pseudotime to identify genes that were down/upregulated early versus late in the process (Figure 5A and S5A; Table S3). Individual genes were clustered based on similarity of their expression trends along pseudotime. We noticed that pluripotency-associated genes followed distinct trends of early, gradual or late upregulation. In agreement with our flow cytometry data, *Oct4* was among the early activated genes, along with *Rex1*, *Mybl2*, *Crxos*, *Egr1* and *Fgf4* (Cluster 0), while other pluripotency regulators such as *Nanog* and *Esrrb* (Clusters 8 and 5, respectively) were only upregulated at the final stages, suggesting the presence of barriers to their activation. In contrast with the asynchronous activation of pluripotency-associated genes, the endoderm-related transcriptional program was silenced in a slow but coordinated manner with an initial downregulation around the T2 stage with complete silencing only achieved during final stages of reprogramming. We therefore hypothesized that dismantling the XEN network was a critical milestone for successful XEN-to-iPS reprogramming. Accordingly, flow sorted cells having lost PDGFRα expression (S-O+P-) at day 14 were able to generate stable, transgene-independent iPS-like cells, when plated in serum/LIF medium in the absence of dox and AGi, while cells with persistent PDGFRα expression (S-O+P+) failed to do so (Figure S5B).

To directly test the inhibitory effect of the XEN program on XEN-to-iPS conversion, we knocked out *Gata4* and determined the impact on XEN-to-iPS reprogramming. GATA4 is a potent inducer of the XEN state in ES cells and required for PrE lineage differentiation^22–27^. CRISPR-Cas9 knockout of *Gata4* (Figure 5B-C and S5C) in XEN cells prior to reprogramming resulted in significantly increased reprogramming efficiency (>7-fold) as assessed by the relative proportion of S+O+P- cells in culture at day 10 of reprogramming (Figure 5D). To gain insights into the molecular basis of increased XEN plasticity upon GATA4 depletion, we performed RNA-seq at early reprogramming stages (day 10 of dox induction) comparing *Gata4* KO to empty vector control cells (Figure 5E). Differential gene expression analysis showed a large number of up- and downregulated genes (Figure 5F; Table S3), strongly associated with developmental processes (Figure S5D). Importantly, ~75% of downregulated DEGs overlapped with the cluster 4 gene set identified from the scRNA-seq gene expression trends (shown in Figure 5A), which predominantly included PrE-related genes resistant to silencing during XEN-to-iPS transition (Figure 5G). *Gata4* KO also caused significant – albeit moderate (~2–4 fold) – upregulation of pluripotency-associated genes (belonging to clusters 0, 8, 5 and 1 in Figure 5A), including those that were upregulated late in the reprogramming process (clusters 5 and 1) (Figure 5G-H). We further generated GATA4 and GATA6 CUT&RUN datasets from starting XEN cells, which identified the majority of downregulated genes upon *Gata4* KO as likely direct GATA targets – ~64% overlapped with at least one C&R peak within 50kb from TSS, whereas only ~45% of upregulated genes showed such overlap (similar to non-DEGs), suggesting indirect activation, for example by repressing an inhibitor (Figure S5E-F). Together, these data demonstrate that GATA4 plays a critical role in safeguarding PrE identity and counteracting OKS activity towards iPS generation. GATA4 expression and activity is therefore a major bottleneck for XEN-to-iPS reprogramming and contributes to the limited plasticity of XEN cells.

### Remodeling to an EPI-like chromatin state constitutes another roadblock during XEN-to-iPS reprogramming

Another explanation for the drastically different efficiencies of XEN and ES interconversions could be due to differential epigenetic plasticity. ATAC-seq analysis in bulk ES and XEN cells, showed a significantly higher proportion of loci exhibiting open chromatin in ES cells compared to XEN cells (49625 versus 12670, respectively), suggesting the ES genome is more accessible than the XEN genome (Figure 5I). Additionally, we noted that several XEN-associated loci (XEN and T1 signature genes based on the scRNA-seq analysis) showed similar levels of accessibility in XEN and ES cells, while iPS/ES-associated loci (iPS signature genes) exhibited significantly lower accessibility in XEN cells (Figure 5J and S5G). Notably, genes associated with the T2 state also showed reduced accessibility in XEN cells, compared with ES/iPS cells. Based on these observations, we wondered whether chromatin remodeling towards a more open and plastic EPI state could represent another roadblock impacting XEN reprogramming.

**Figure 6.**
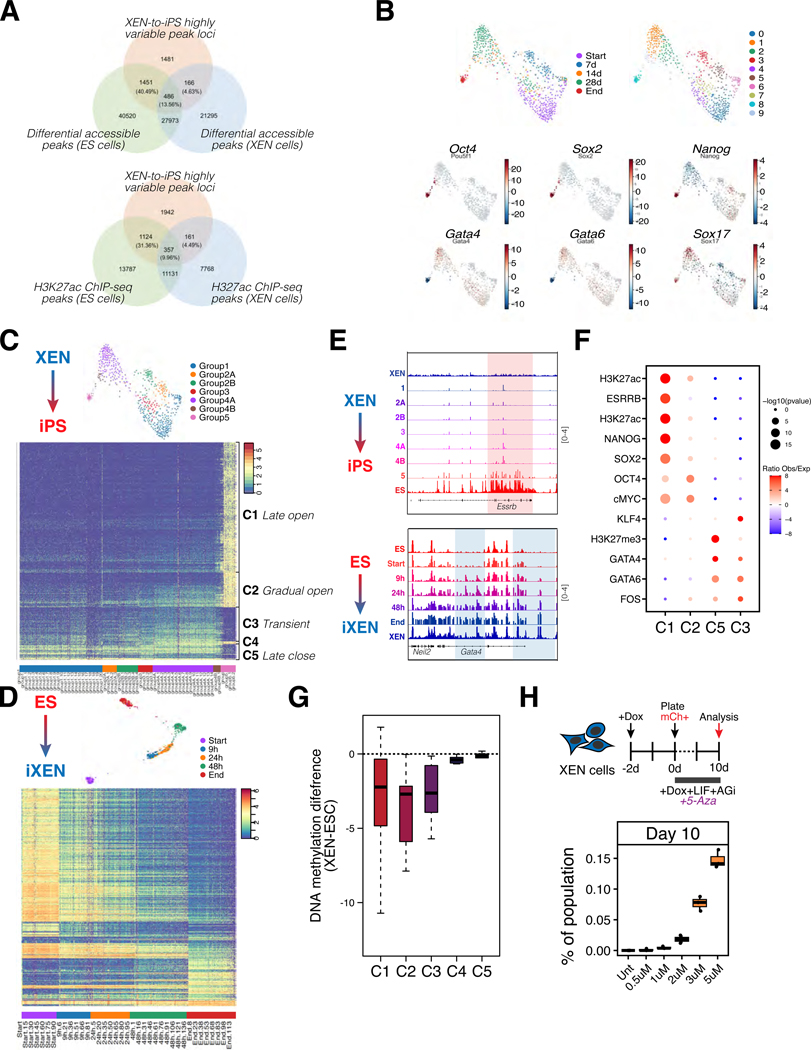
Establishing an EPI-like chromatin state underlies the inefficient XEN-to-iPS conversion. **(A)** Venn diagrams depicting number and percentages of highly variable accessible peak loci identified from scATAC-seq data for XEN-to-iPS conversion trajectory that overlap with differential accessible peak loci identified from bulk ATAC-seq data for XEN and ES cells *(top)*, or with H3K27ac ChIP-seq peak loci in XEN and ES cells *(bottom)*. **(B)** Force-directed layouts of combined scATAC-seq dataset for XEN-to-iPS conversion, highlighting individual timepoints *(top left)*, metacell clusters *(top right)*, or ChromVAR scores for XEN- and ES-specific TFs *(bottom)*. Number of cells = 61,040; number of metacells = 543. **(C)** *(Top)* Force-directed layout of XEN-to-iPS conversion scATAC-seq dataset highlighting the groups of metacells identified based on similar accessibility profiles along pseudotime. *(Bottom)* Heat map view of relative chromatin accessibility changes over pseudotime of XEN-to-iPS conversion for a subset of selected peak loci, from group 1 to group 5 of the metacells. Chromatin accessibility is measured in units of number of fragments (median-normalized) across metacells followed by log transformation with a pseudo-count of 1. Each row corresponds to a peak locus and each column corresponds to a metacell. Peak loci shown are differential accessible peak loci identified in the XEN-to-iPS conversion based on differential accessibility analysis of peak loci between group 4B and group 5 of the metacells. **(D)** *(Top)* Force-directed layout showing the scATAC-seq metacell data for ES-to-iXEN conversion and highlighting the individual timepoints (number of cells = 95,396; number of metacells = 567). *(Bottom)* Heatmap view of the relative chromatin accessibility changes of ES-to-iXEN conversion based on the highly variable peak loci that overlap between the ES-to-iXEN and XEN-to-iPS conversions. **(E)** Representative IGV (Integrative Genomics Viewer) tracks showing accessibility peaks of pseudo-bulk scATAC-seq data of XEN-to-iPS *(top)* or ES-to-iXEN *(bottom)* conversion. Highlighted are relative accessibility in metacell groups (top panel; XEN-to-iPS) or individual timepoints (bottom panel; ES-to-iXEN) at example genomic loci showing late opening in XEN-to-iPS, versus gradual opening in ES-to-iXEN conversion. Signal values are indicated to the right. **(F)** LOLA enrichment analysis of late opening, gradual opening, late closing or transient opening peaks during XEN-to-iPS conversion. **(G)** Boxplots showing differences in DNA methylation levels between XEN and ES cells of highly variable loci depicted in (C). **(H)** XEN reprogramming with different doses of 5-Azacytidine. Box plots show the proportion of iPS-like cells (S+O+P-) at day 10 for reprogramming. N = 3.

We therefore performed single-cell ATAC-seq (scATAC-seq) to quantify dynamic accessibility changes during each lineage conversion (XEN-to-iPS and ES-to-iXEN). We sampled ~100,000 nuclei from the same timepoints as in our scRNA-seq dataset, and generated ‘metacells’ using the SEACells algorithm^68^. Individual metacells represent small groups of single cells (110 on average) with similar accessibility states. We focused our analysis of the XEN-to-iPS conversion on loci with highly variable accessibility among metacells (N=3584; Table S4). A large fraction of these loci (40.49%) represented ES-specific peaks (compared to only 4.63% overlapping with XEN-specific peaks) as detected by bulk ATAC-seq (presented in Figure 5I) and overlapped predominantly with putative enhancers of ES cells compared with XEN, as detected by bulk H3K27ac ChIP-seq (31.36% versus 4.49%, respectively) (Figure 6A). These results indicate that XEN-to-iPS reprogramming is accompanied by extensive chromatin opening around EPI/ES-related regulatory regions.

ChromVAR analysis^69^ depicting accessibility changes around the motifs of key EPI or XEN-related regulators, suggested that key cell identity-related changes occurred mostly during the final stages of reprogramming. Notably, motifs for OCT4 and SOX2, despite their continuous exogenous expression, displayed high accessibility only at late stages of XEN-to-iPS reprogramming (Figure 6B). For a genome-wide analysis of the kinetics and potential drivers of accessibility changes, we performed PhenoGraph^70^ clustering of metacells undergoing XEN-to-iPS conversion, which assigned 7 major groups (1, 2A, 2B, 3, 4A, 4B and 5) based on relative accessibility during conversion (Figure 6C). A heatmap of the most variable peaks (N=3584; Table S4) across the 7 groups showed extensive and slow chromatin opening and rather restrictive closing during XEN-to-iPS conversion. On the other hand, tracking the most variable peaks during the opposite ES-to-iXEN transition (N=1955 that overlap with the highly variable peaks of the XEN-to-iPS trajectory; Table S4) showed more rapid and progressive changes (Figure 6D-E and S6A-C), consistent with the efficient nature of this conversion. Enrichment analysis using published ChIP-seq datasets^71^ showed a significant association for known regulators of EPI (e.g. NANOG, OCT4, SOX2, ESRRB) or PrE fate (e.g. GATA4, GATA6) among the opened or closed regions, respectively (Figure 6F; Table S5). However, we also detected a subset of regions that opened more gradually in the XEN-to-iPS trajectory, as well as a group of transiently accessible regions that only lost accessibility once pluripotency was established (Figure 6C). Gradually opening peaks were enriched for binding of OCT4 and MYC, while transiently open peaks were enriched for GATA factors (PrE program) and FOS (AP-1 complex) binding, and overlapped with sites silenced by H3K27me3 histone marks in ES cells. This could suggest that the persistent expression of GATA factors might derail reprogramming by opening up new sites toward alternative or dead-end states. Together, these data highlight that many critical EPI and PrE regulatory elements are refractory to chromatin remodeling during the XEN-to-iPS conversion, likely contributing to this being a slow and inefficient process.

To better understand the molecular basis of the slow and inefficient chromatin opening during XEN-to-iPS conversion, we integrated published DNA methylation datasets from XEN and ES cells^54^. Our analysis showed that regions gaining chromatin accessibility during XEN-to-iPS reprogramming (either gradually, transiently or at late stages) had substantially higher DNA methylation levels in XEN compared to sites that were initially open and decommissioned during reprogramming (Figure 6G and S6D). Therefore, chromatin opening during XEN-to-iPS conversion associates with a significant loss of DNA methylation, while chromatin closing involves no substantial methylation changes. This finding combined with the gradual or late activation of *Tet2* and *Tet1* enzymes, respectively, according to our scRNA-seq analysis (see Figure 5A, clusters 0 and 8) suggested that DNA methylation could represent an epigenetic barrier during XEN reprogramming. In agreement, XEN cells treated with 5-Azacytidine during reprogramming showed a strong, dose-dependent increase in their reprogramming efficiency (Figure 6H). In conclusion, these data presented two additional molecular roadblocks during the inefficient and slow XEN-to-iPS conversion: the requirement for extensive chromatin opening and DNA demethylation.

## DISCUSSION

The EPI and PrE lineages of the early mammalian embryo arise from a common ICM progenitor, and thus have a unique lineage relationship, the molecular basis of which remains poorly understood. In this study we probed the relative plasticity of these two sister lineages and determined whether cells in the PrE state can revert to an EPI/pluripotent state by manipulating the transcription factor network. We took advantage of mouse stem cells representing these lineages *in vitro* – ES (EPI) and XEN (PrE) cells – representing a scalable and tractable system for analysis. By establishing a bidirectional conversion system between XEN and ES cells by ectopic expression of either *Oct4*, *Klf4* and *Sox2* (OKS) or of *Gata4*, we demonstrate for the first time a marked difference in the relative plasticity of the two lineages. While ES cells can be efficiently converted to iXEN cells, over ~4 days and >95% efficiency, XEN cells reprogram to iPS cells with an extremely low efficiency, requiring ~3 weeks and ~0.2% efficiency.

The slow and inefficient reprogramming of XEN-to-iPS cells was notable for several reasons. First, OKS(M) reprogramming of numerous cell types has displayed an inverse correlation between the differentiation status and reprogramming amenability of a cell^72,73^. Similarly, transdifferentiation experiments suggest that cells with closer developmental relationships interconvert more readily^74–76^. Therefore, XEN cells derived from a common progenitor as the EPI might be expected to have a relatively high potential for acquiring an EPI/iPS state. In line with this reasoning, chemical reprogramming of somatic cells to iPS cells, suggested that a XEN-like state showed increased plasticity, permitting reprogramming to pluripotency^28–30,77^. Although, a recently modified chemical reprogramming protocol with improved kinetics does not require a XEN-like transition state for efficient generation of human iPS cells^78^. Moreover, XEN cells divide rapidly in culture, a feature that is associated with increased reprogramming efficiencies^79,80^. Despite these favorable properties, XEN cells showed a surprisingly low reprogramming potential, indicating an unusually stable state, which resists acquisition of pluripotency. Accordingly, scRNA-seq analysis identified at least two major bottlenecks that XEN cells need to overcome during reprogramming. The first involves activation of key pluripotency genes, such as *Oct4* and *Rex1*, a common roadblock for most somatic cell types. The second requires downregulation of key XEN-associated genes, and appears unique to this cell type, since silencing of the somatic program is usually the first and most efficient milestone during reprogramming of somatic cell types^48,81–83^. These observations highlight the unusual stability of the XEN/PrE program which cannot be easily overwritten by the potent Yamanaka factors (OCT4, SOX2, KLF4). This is partly due to a tight transcriptional network governed by the GATA factors, as perturbing GATA4 expression significantly increased reprogramming efficiency. Moreover, transient expression of *Gata4* in iPS cells was sufficient to generate iXEN cells with reduced plasticity that are largely refractory to reprogramming. Our bulk and single-cell ATAC-seq datasets also noted a more rigid epigenetic/chromatin state in XEN that requires extensive remodeling to establish a pluripotent program. The less favorable chromatin state of XEN compared to ES cells, especially around critical EPI and PrE gene loci, has been reported before at the level of DNA methylation and histone modifications^54,55,84^. Indeed, treatment of XEN cells with 5-Azacytidine significantly increased their reprogramming capacity, pinpointing DNA methylation as a major epigenetic bottleneck. Furthermore, our scRNA-seq analysis showed somewhat divergent transcriptional changes during reprogramming in either direction, characterized by differential expression of chromatin modifiers and genes regulating cellular metabolism in transitional stages. These findings reflect reported epigenetic differences, as well as metabolic differences between the two lineages^56–58^. Overall, our findings suggest that the EPI and PrE fate bifurcation is accompanied by drastic epigenetic and metabolic changes, posing a challenge for lineage conversion going from XEN to ES, but not vice versa.

In further relating the *in vitro* states to the EPI and PrE lineages *in vivo,* we found that the *in vitro* trajectories approximated sequential cell states in the embryo, mapping to the EPI and PrE lineages, but bypassing the uncommitted ICM progenitor state, indicating that this system is likely bistable, with the ICM perhaps representing an unstable *in vivo* state. This suggests that ICM lineage specification is a dynamic process with cells not occupying an uncommitted state in the absence of extracellular signals^26,66,85^. Notably, reprogramming XEN cells tracked along the PrE trajectory from the point of PrE lineage commitment through to upregulation of later PrE genes, such as *Gata4* and *Sox17*, in the blastocyst^6,86^. We therefore hypothesize that more ‘PrE-like’ *in vitro* states would be more amenable to reprogramming to an iPS state. One such state identified as the ‘T2’ terminal state in our scRNAseq dataset co-expresses endoderm genes and a subset of pluripotency-associated genes, and displays higher differentiation potential than the starting XEN state. Indeed, our flow cytometry data showed that *Oct4*-GFP+ PDGFRα+ intermediate cells represent a more advanced reprogramming state than the starting (Oct4-GFP-PDGFRα+) XEN population.

In conclusion, our work clearly demonstrates the differential plasticity of ES and XEN cells and offers insights into its molecular basis. Further studies will likely determine the functional and developmental relevance of epigenetically restricting the PrE lineage soon after specification. Extra-embryonic cells tolerate polyploidy to a greater degree than cells of the embryo-proper, and also display unique genomic imprinting patterns^87–91^. A parsimonious explanation could be to restrict mixing of embryonic and extra-embryonic compartments and prevent “unfit” cells from disrupting development of the embryo-proper and establishment of the germ line. Lineage switching and subsequent mixing in distinct tissue layers *in vivo* might be deleterious to key developmental processes. Segregating embryonic and extra-embryonic compartments organizes robust and reproducible signaling environments directing fate specification and morphogenetic events during gastrulation.

### Limitations of the Study

Our study demonstrates differential plasticities of ES and XEN cell states, their potential for interconversion and molecular underpinnings of their differences. However, there are some limitations that warrant discussion. First, we present here a singular mode of TF-mediated lineage conversion in either direction from starting XEN or ES cells. Alternative TF cocktails and/or reprogramming medium compositions that modulate cellular signaling, for example, would need to be tested with regards to their impact on interconversion efficiencies. A deeper characterization of refractory and transitional states during XEN-to-ES conversion could identify specific factors or signaling pathways to target. Second, our analysis of the *in vitro* lineage conversion trajectories and comparison to *in vivo* cell states indicates that *in vitro* cells with a more naïve ‘PrE-like’ character would have greater reprogramming potential than XEN cell. Subsequent studies would test this directly, identifying appropriate markers to isolate different intermediate states along the XEN-to-iPS trajectory and assessing their reprogramming potential. Furthermore, additional characterization and comparison of alternative PrE *in vitro* stem cell models (e.g. nEnd cells^62,63^ and PrESCs^64)^, and integration with our single-cell and bulk RNA-seq and ATAC-seq datasets would serve to test this hypothesis.

## RESOURCE AVAILABILITY

### Lead contact

Further information and requests for resources and reagents should be directed to the lead contact, Anna-Katerina Hadjantonakis (hadj@mskcc.org).

### Materials availability

pSpCas9(BB)-2A-GFP (PX458) was a gift from Feng Zhang (Addgene plasmid # 48138; http://n2t.net/addgene:48138; RRID:Addgene_48138).

Cloning methods for other plasmid constructs based on the available plasmids are described in the methods details section. All materials generated in this study are available upon request from the lead contact.

### Data and code availability

The various datasets generated in this study have been deposited in the National Center for Biotechnology Information (NCBI) Gene Expression Omnibus (GEO) under the series accession numbers GSE266451, GSE266452, GSE266453, GSE276702, GSE276704 and are publicly available as of the date of publication. Accession numbers are listed in the Key Resources Table. All data reported in this paper and any additional information required to reanalyze the data are available from the lead contact upon request.

## STAR METHODS

### EXPERIMENTAL MODEL AND STUDY PARTICIPANT DETAILS

#### Mouse strains and husbandry conditions

All animal work was approved by Memorial Sloan Kettering Cancer Center’s (MSK) Institutional Animal Care and Use Committee, protocol 03–12-017 (Hadjantonakis PI), and Weill Cornell Medicine’s (WCM) protocol 2014–0044 (Apostolou, PI). Animals were housed in a pathogen-free facility under a 12-hr light cycle, with daily monitoring, and food and water available *ad libidum*. All embryos used for this study were obtained from natural matings of virgin females of 5–10 weeks of age. Euthanasia was performed by CO2 as recommended by the 2007 AVMA Guidelines on Euthanasia and approved by MSK and WCM IACUCs. Mouse strains used in this study were: *Col1A1*^*TetO-OKSmCh/TetO-OKSmCh*^; *R26*^*M2rtTA/M2rtTA*^ (Jackson Labs, Bar Harbor, ME, USA/stock ID: 034917)^33^, *Oct4-GFP* (Jackson Labs, Bar Harbor, ME, USA/stock ID: 008214)^36^, and wild-type CD1 (Charles River) or C57BL/6 (Jackson Labs, Bar Harbor, ME, USA/stock ID: 000664). Genotyping PCR bands are as follows: *Col1A1*^*TetO-OKSmCh*^: wild-type – 331bp, knock-in – 551bp; *R26*^*M2rtTA*^: wild-type – 500bp, knock-in – 250bp; *Oct4-GFP*: wild-type – 434bp, knock-in – 234bp. Primers for genotyping are listed in supplemental Table S6.

#### Murine cell lines, derivation and culture

XEN cell lines used in this study were: IM8A-1^11^ and reprogrammable ‘3F’ XEN lines. 3F XEN cells were derived from mice harboring *Col1a1*^*TetO-OKSmCh*^, *R26*^*M2rtTA*^ and *Oct4-GFP* alleles using either TS cell conditions (3F4 line) or ES cell conditions (3F2, 3F6 and 3F9 lines) as detailed elsewhere^111^. Established XEN and iXEN cells were cultured in standard XEN cell culture conditions^11,111^. Cells were seeded onto tissue culture grade plates coated with 0.1% gelatin (Millipore Sigma) for 5 mins at room temperature. Roswell Park Memorial Institute (RPMI) 1640 (Gibco) or Dulbecco’s modified Eagle’s medium (DMEM; Gibco) was supplemented with 15% FBS, 2 mM L-glutamine (Gibco), 1 mM sodium pyruvate (Gibco), 100 U/ml Penicillin, 100 μg/ml Streptomycin (Penicillin-Streptomycin; Gibco) and 0.1 mM 2-mercaptoethanol (Gibco).

ES cell lines used in this study were: R1 ES cells^37^, v6.5 ES cells^93^ and *Col1a1*^*TetO-Gata4-mCherry/+*^;*R26*^*M2rtTA/+*^;*Gata6*^*H2B-Venus/+*^ ES cells^24,44^. ES cells and XEN-iPS cells were cultured in standard serum/LIF conditions as described previously^112^. Cells were plated onto tissue culture grade plates coated with 0.1% gelatin for 5 mins at room temperature. DMEM was supplemented with 15% FBS, 0.1 mM non-essential amino acids (NEAA; Gibco), 2 mM L-glutamine, 1 mM sodium pyruvate, 100 U/ml Penicillin, 100 μg/ml Streptomycin, 0.1 mM 2-mercaptoethanol and 1000 U/ml leukemia inhibitory factor (LIF; prepared in house).

All cells were passaged every 2 days (~80% confluence) by washing with phosphate buffered saline (PBS) followed by brief incubation in 0.05% Trypsin-EDTA (Gibco) at 37°C ~2–3 mins). Trypsin activity was neutralized with serum-containing media (3x volume of Trypsin used) and dissociated cells were centrifuged at 400*g* for 3 mins before resuspending in culture media. Cells were replated at 1:8–1:10 dilution.

### METHODS DETAILS

#### XEN-to-iPS and ES-to-iXEN conversions

For XEN-to-iPS conversion, reprogrammable XEN cells were first cultured in standard XEN cell media with 2 μg/ml doxycycline (‘dox’; MP Biomedicals) for 2 days. mCherry-positive XEN cells were sorted using flow cytometry (see below) and plated onto mitomycin C-treated mitotically inactivated MEF feeders in 6-well or 10 cm plates for reprogramming. All plates were pregelatinized and layered with approximately 1×10^6^ MEF feeders per 50 cm^2^ culture surface area. mCherry-sorted XEN cells were reprogrammed in standard serum/LIF media supplemented with 2 μg/ml dox, 50 μg/ml ascorbic acid (Millipore Sigma) and 3 μM GSK3βi/CHIR99021 (Reprocell) (‘AGi’ media)^33^. Media was replaced every two days. Doxycycline and ascorbic acid were prepared fresh every 5–7 days in dH_2_O and stored at 4°C protected from light. Doxycycline was filter sterilized using a 0.2 μm SFCA filter (Thermo Scientific). Reprogramming cells were dissociated with 0.25% Trypsin-EDTA prior to downstream analysis (Gibco).

ES-to-iXEN conversion was carried out as described previously^25^. ES cells were plated onto pregelatinized plates in standard serum/LIF media supplemented with 2 μg/ml dox. Media was replaced every two days. Cells were dissociated with 0.05% Trypsin-EDTA prior to downstream analysis.

Supplemental Table S6 outlines the XEN and ES cell numbers plated for reprogramming.

#### iPS-to-iXEN conversion

A *pCX-Gata4-E2A-E2-Crimson* expression vector was first generated using a *pCAGGS* vector^113^ as a backbone digested with EcoRI restriction enzyme. Coding sequences for mouse *Gata4* and *E2-Crimson* preceded by an E2A self-cleaving peptide (*E2A-E2-Crimson*) were assembled using NEBuilder HiFi DNA assembly (NEB) to generate *pCX-Gata4-E2A-E2-Crimson*. iPS cells derived from ProB cells from mice harboring *Col1a1*^*TetO-OKSmCh*^, *R26*^*M2rtTA*^ and *Oct4-GFP* alleles were transfected with *pCX-Gata4-E2A-E2-Crimson* plasmid using Lipofectamine 3000 (Invitrogen). *Oct4*-GFP+/PDGFRα+ and Oct4-GFP-/ PDGFRα+ cells were flow sorted on Day 5 or Day 7 of conversion, then plated onto XEN-to-iPS reprogramming conditions and analyzed for marker expression 14 days following sorting and plating. In parallel, freshly converted iXEN cells (passage 2 following iXEN formation) from ProB iPS cells were also plated and analyzed for marker expression by flow cytometry.

#### Immunophenotyping by flow cytometry and fluorescence activated cell sorting

Dissociated cells were filtered through a 0.35μm nylon mesh strainer (Falcon) to achieve a single cell suspension. Cells were centrifuged at 400*g* for 5 mins at room temperature and washed twice with wash buffer (5% FBS in PBS without Ca^2++^ and Mg^2++^). Cell pellets were then resuspended in 100 μl staining buffer (wash buffer with diluted antibodies) and incubated at room temperature for 30 mins protected from light. Following incubation, cells were washed twice with wash buffer. Cell pellets were finally resuspended in wash buffer containing 1μg/ml 4′,6-diamidino-2-phenylindole (DAPI; Invitrogen) or 7-AAD viability dye (BioLegend; 1μl/sample). The following antibodies were used per 100μl of staining volume for 1×10^6^ cells: PE-Cy7::CD140a (PDGFRα; eBioscience) at 0.0625μg/μl, AlexaFluor647::SSEA-1 (BioLegend) at 2.5μl/sample or BV421::SSEA-1 (BioLegend) at 2.5μl/sample. Stained cell samples were then analyzed for marker expression using LSRFortessa (BD Biosciences). DAPI and BV421 were excited at 405 nm and detected using 450/50 nm band-pass filters. GFP and Venus were excited at 488 nm and detected using 525/50 nm band-pass filter. mCherry was excited at 561 nm and detected using 610/20 nm band-pass filter. 7-AAD was excited at 561 nm and detected using 670/30 nm band-pass filter. PE-Cy7 was excited at 561 nm and detected using 780/60 nm band-pass filter. AlexaFluor647 was excited at 633 nm and detected using 670/30 nm band-pass filter. For cell sorting, samples were prepared as described and resuspended at a final concentration of 5–10×10^6^/ml in wash buffer. Cells were sorted using SORP FACSAria IIu (BD Biosciences) with a 100μm nozzle at 20psi. Gating strategies are provided in Figure S2A. Data were analyzed using FlowJo and R (http://www.r-project.org/).

#### Real-time quantitative PCR

Cells were harvested following trypsinization and washed once with PBS. Total RNA was extracted from cells pellets using TRIzol reagent (Invitrogen) according to manufacturer’s directions. cDNA synthesis was carried out with 1 μg of RNA using the QuantiTect Reverse Transcription Kit (Qiagen) and subsequently diluted 1:25 in dH_2_O. 5 μl of resulting cDNA was combined with 1 μM each of forward and reverse primers and 10 μl of PowerUp SYBR Green Master Mix (Applied Biosystems) for RT-qPCR in 20 μl of total volume. RT-qPCR reactions were carried out in a CFX96 Real-Time PCR detection system (BioRad). All analyses were carried out in R. Raw Ct values of three technical replicates per reaction were averaged and normalized to the mean of two reference genes: *Actb* and *Gapdh*. Normalized Ct values were then plotted as “expression” values using the following equation: *y* = 2^*-Ct*^. The primers used are listed in Supplemental Table S6.

#### Immunofluorescence

##### Cultured cells.

Cells were washed twice for 5 mins each with PBS before fixation at room temperature with 4% PFA (Electron Microscopy Sciences) for 15 mins. Fixed cells were washed again with PBS twice for 5 mins each before permeabilization with PBST [PBS + 0.1% Triton-X 100 (Millipore Sigma)] at room temperature for 10 mins. Cells were subsequently blocked with blocking buffer [PBST + 1% bovine serum albumin (BSA; Millipore Sigma) + 3% donkey serum (Jackson ImmunoResearch)] at room temperature for 15 mins. Cells were then incubated with primary antibodies diluted in blocking buffer overnight at 4°C. Following incubation, cells were washed three times for 10 mins each with PBST. Cells were then incubated for 2 hrs with secondary antibodies diluted in blocking buffer at room temperature. Next, cells were washed twice for 10 mins each with PBST before incubation with DAPI diluted 1:10,000 in PBST for 10–15 mins. DAPI was washed off with PBS before imaging.

##### Post-implantation stage embryos.

Freshly dissected embryos were fixed in 4% PFA at room temperature for 15 mins. Following fixation, embryos were washed once with PBST before permeabilization in 0.5% Triton-X (in PBS) at room temperature for 30 mins. Fixed and permeabilized embryos were then blocked with blocking buffer (PBST + 1% BSA + 5% donkey serum) overnight at 4°C. Next, embryos were incubated with primary antibodies diluted in PBST + 1% BSA overnight at 4°C. The following day, embryos were washed 3 times for 10 mins. each in PBST. Embryos were then blocked for 5 hrs at room temperature prior to incubation with secondary antibodies diluted in blocking buffer overnight at 4°C. Stained embryos were then washed twice with PBST for 10 mins each before incubation with DAPI diluted 1:1000 in PBST. Embryos were finally washed in PBS before imaging.

Secondary AlexaFluor antibodies (donkey/IgG; Invitrogen) were used at 1:500 dilution. See Key Resources Table for list of primary antibodies used in this study.

#### XEN-iPS cell-embryo chimeras

Prior to generating chimeric embryos, XEN and XEN-iPS cells were labeled with an *mCherry* expression vector. For this, a *pCX-mCherry* expression vector (unpublished) was used in which *mCherry* is inserted into a *pCAGGS* vector^113^ as previously described^114,115^. The plasmid was linearized by *ScaI* restriction digest and purified using the QIAquick PCR purification kit (Qiagen). Purified linear plasmid was transfected using Lipofectamine 3000 (Invitrogen). Stably expressing mCherry-positive XEN cells were flow sorted, and mCherry-positive XEN-iPS clones were picked 10 days following transfection and expanded prior to 8-cell morula injection.

XEN-iPS chimera embryos were generated by the Mouse Genetics Core at MSKCC. XEN or XEN-iPS cells were injected into C57BL/6 host 8-cell morula, and transferred to pseudo-pregnant females. Chimeric embryos were recovered at E5.5-E6.0.

#### Genetic editing of XEN cells

To knockout *Gata4* or *Gata6* gene expression in XEN cells, the PX458 vector (Addgene #48138) was first modified to express E2-Crimson instead of EGFP. The resulting PX458-E2-Crimson vector was digested using BbsI-Hf (NEB) and single guide RNA (sgRNA) targeting *Gata4* or *Gata6* was annealed as previously described^116^. Top 3 ranked sgRNAs were tested from the CHOPCHOP tool^117^. Assembled Cas9/sgRNA plasmids were transfected into XEN cells using Lipofectamine 3000 (Invitrogen). E2-Crimson-positive cells were assessed for successful knockout of gene expression and reprogramming potential (see Figure 5C and S5C). pSpCas9(BB)-2A-GFP (PX458) was a gift from Feng Zhang (Addgene plasmid # 48138; http://n2t.net/addgene:48138; RRID:Addgene_48138).

#### Image acquisition and processing

Brightfield and epifluorescence images shown in Figure 1A-B were acquired on a Zeiss Axio Vert.A1 inverted microscope with a black and white camera (Axiocam MRm). Brightfield and epifluorescence images shown in Figure S1E were acquired on a Zeiss AxioZoom stereomicroscope with a Zeiss Axiocam MRc CCD camera and ZEN 2.3 software, using the manual extended depth of focus application. Immunofluorescence images shown in Figure 1E, S1B and S5C were acquired on a Zeiss LSM 880 laser-scanning confocal microscope. Post-implantation stage embryos were imaged in a microdrop of PBS on a 35 mm glass bottom dish (MatTek) using a Plan-Apo 20×/NA0.8 M27 objective. Z-stacks were taken at 0.88-μm intervals. Images in Figure S1B and S5C were acquired using an EC Plan-Neofluar 40×/NA1.30 oil immersion objective at 1-μm z-intervals. Fluorescence was excited using a 405-nm diode (Hoechst 3342), 488-nm argon, 561-nm DPSS-561–10 and HeNe 633-nm lasers. Raw image data were processed using ImageJ (Rasband, W.S., ImageJ, U.S. National Institutes of Health, Bethesda, Maryland, USA, https://imagej.nih.gov/ij/, 1997–2018). Raw images for Figure S5C were processed using Imaris (Bitplane), where nuclei were identified using the Spot model, and corresponding relative fluorescent intensities were measured for each channel. Data were analyzed using R (http://www.r-project.org/).

#### Bulk RNA-seq analysis

RNA-seq libraries were prepared and sequenced by Novogene Bioinformatics Technology Co. Ltd. In brief, total RNA was extracted from 1×10^6^ cells per duplicate using TRIzol reagent (Invitrogen), and genomic DNA was removed. RNA concentrations were measured using a Qubit RNA Assay Kit with a Qubit 2.0 Fluorometer (Thermo Scientific). A total of 3μg RNA per sample was used for sample preparations and library constructions. Then, ribosomal RNA was removed using the Illumina Ribo-Zero rRNA Removal Kit (Illumina), and rRNA-free residue was discarded by ethanol precipitation. Sequencing libraries were prepared using the NEBNext Ultra Directional RNA Library Prep Kit for Illumina (New England Biolabs) following the manufacturer’s instructions. High-throughput sequencing was performed on an Illumina NovaSeq 6000 platform.

##### Analysis for ES, XEN and XEN-iPS cells:

Paired-end sequenced reads from ES, XEN and XEN-iPS cell lines were aligned to mouse genome (mm10) with Tophat2 (version 2.1.1) with default setting and “-r 200 –mate-std-dev 100” option. Sorting of aligned reads was performed with samtools^95^ and reads were assigned to protein coding and long-non coding genes (Mus_musculus.GRCm38.95.gtf) with the use of htseq-count^96^ and ‘-m intersection-nonempty’ option. DESeq R package^97^ was used to call differentially expressed genes between XEN and ES cell lines. Only genes with p-adj (<0.01) and fold change cut off (2) were considered as differentially expressed between ES and XEN.

##### Analysis for Gata4 KO samples:

Paired-end read alignment to mouse genome (mm10 version) was performed with STAR aligner (version 2.7.10)^99^ with default setting. Samtools^95^ were used for filtering and sorting aligned reads before annotation to “mm10.GRCm38.95.gtf” gene version with featureCounts (subread 2.0.6 version)^100^. Only protein-coding and long-non-coding RNA transcripts were used for annotation and downstream differential expression analysis was performed with R package DESeq2^98^ where we set a log2 fold change of 1.5 and P-adjusted of <0.05 as a cut off for calling deferentially expressed genes.

#### Sample preparation for single-cell RNA-seq and single-cell ATAC-seq

XEN-to-ES and ES-to-XEN conversions were carried out as described for the durations outlined in Figure 3A prior to collection. For ‘starting’ populations, XEN cells were cultured on MEF feeders for 7 days in reprogramming media without dox induction (i.e., AGi media minus dox), and ES cells were cultured in standard serum/LIF conditions prior to collection. To collect nascent XEN-iPS cells, XEN cells were reprogrammed for 14 days, sorted for SSEA-1- GFP+ PDGFRα- cells using flow cytometry (see above) and replated for an additional 14 days (day 28 of reprogramming). At this point SSEA-1+ GFP+ PDGFRα- cells were sorted and replated onto MEF feeders in standard serum/LIF media for 7 days prior to dissociation. A similar strategy was applied for nascent iXEN cells – ES cells were treated with dox for a period of 4 days, by when >95% of cells have successfully converted to iXEN (see Figure 2). Following this period, dox was withdrawn from the culture media for 7 days prior to collection. As described above and schematized in Figure S2E, a cell sorting strategy was used for XEN-to-iPS converted day 7, day 14 and day 28 cells, and ES-to-XEN converted 9h cells. ES-to-XEN 24h and 48h samples were not sorted.

All cells were dissociated using either 0.05% or 0.25% Trypsin-EDTA and dissociated into single cells by repeated pipetting and passing through a 0.35 μm nylon mesh strainer prior to downstream processing. For scRNA-seq samples, dissociated or sorted cells were counted and diluted in DMEM + 10% FBS. Final cell suspensions were loaded on a Chromium Controller targeting a 5,000 – 8,000 cell range, depending on the sample, to generate single-cell 3’ RNA-seq libraries in duplicate^118^. For scATAC-seq samples, cells were washed in PBS + 0.04% BSA and processed for nuclei isolation following manufacturer’s instructions. Final nuclei preparations were loaded on a Chromium Controller targeting 10,000 nuclei, and processed in singlicate. Libraries were generated following the manufacturer’s instructions (10x Genomics Chromium Single Cell 3′ Reagent Kit User Guide v2 Chemistry and 10x Genomics Chromium Next GEN Single Cell ATAC Reagent Kit Guide v1.1).

To obtain single parietal endoderm cells from E7.5 and E8.5 wild-type mouse embryos, we first dissected the embryo out of its decidua, and then teased the parietal yolk sac out the decidua in DMEM/F12, 5% Newborn Calf Serum (Gibco). To remove debris, the tissue was washed five times in 200μl drops of DMEM/F12, 5% Newborn Calf Serum shaking, followed by five washes in DMEM/F12. The parietal yolk sac was then incubated in 100 μl TrypLE (Invitrogen) for 15 min at room temperature for dissociation into single cells. For the subsequent mechanical dissociation 100 μl DMEM/F12, 20% Newborn Calf Serum, 4 mM EDTA was added. The parietal cell clumps were dissociated into single cells first by using a P200 pipette to shake off Reichert’s membrane followed by mouth pipetting with pulled glass capillaries. The resulting single cell suspension was filtered through a FlowMi cell strainers (4 μm, Millipore Sigma) to remove cell clumps and debris and centrifuged, and pellet resuspended into DMEM/F12, 10% Newborn Calf Serum. Cells were stored on ice until loaded onto a Chromium Controller (10x Genomics) targeting 3,000 – 4,000 cells (E7.5), and 9,000 cells (E8.5) to generate single-cell 3’ RNA-seq libraries in duplicate. Of note, our efforts to isolate single parietal endoderm cells from Reichert’s membrane of earlier staged (E5.5 and E6.5) embryos yielded too few cells to load onto a 10x Genomics Chromium chip.

#### Next-generation sequencing of single-cell libraries

Single-cell 3′ RNA-seq and single-cell ATAC-seq libraries were quantified on an Agilent Bioanalyzer with a high-sensitivity chip (Agilent), and Kapa DNA quantification kit for Illumina platforms (Roche). Libraries were pooled according to target cell number loaded for a sequencing depth of 20K-25K (for scRNA-seq) or 35K (for scATAC-seq) reads per cell and accounting for the capacity of an Illumina NovaSeq flow cell. Library pools were loaded on an Illumina NovaSeq 6000 using 2× NovaSeq 6000 S2 reagent kits (200 cycles) and 1× NovaSeq 6000 S4 reagent kits (300 cycles) using the following read length: 26-bp read 1, 8-bp I7 index and 98-bp read 2 (for scRNA-seq), or 50-bp read 1, 8-bp I7 index, 16-bp I5 index and 50-bp read 2 (for scATAC-seq). Libraries of ParE cells isolated from E7.5 and E8.5 embryos were sequenced as previously published^6^.

#### Single-cell RNA-seq data processing

##### Data preprocessing:

scRNA-seq data from each sample was preprocessed using the SEQC pipeline^102^ using GRCm38/mm10 mouse genome and default SEQC parameters to obtain molecule count matrices. The SEQC pipeline aligns the reads to the genome, corrects barcode and unique molecular identifier (UMI) errors, resolves multi-mapping reads, and generates a molecule count matrix. SEQC also performs a number of filtering steps: (1) Identification of true cells from cumulative distribution of molecule counts per barcode, (2) removal of apoptotic cells identified at cells with >20% of molecules derived from the mitochondria, and (3) removal of low-complexity cells identified as cells where the detected molecules are aligned to a small subset of genes. The filtered count matrix was normalized by dividing the counts of each cell by the total molecule counts detected in that particular cell. The normalized matrix was multiplied by the median of total molecules across cells to avoid numerical issues. Normalized data were log transformed with a pseudo-count of 0.1.

##### Data clean-up, dimensionality reduction and visualization:

Each reprogramming trajectory was analyzed separately at first by pooling the two replicates. For each trajectory, 1500 highly variable genes were selected using scanpy^103^. Data was projected onto principal components following gene selection to overcome the noise in scRNA-seq data due to high degree of dropouts. The number of components that explain 85% of the variance were retained for each of the downstream analyses. Force directed layouts were computed for each trajectory by first computing an adaptive kernel^105^ to account for large density differences in the data, computed using the Palantir package^45^ with default parameters. For the XEN-to-iPS dataset, a cluster of cells with fibroblast signature and another with 2-cell stage signature were removed since they are likely feeder and spontaneously differentiated cells respectively. For the ES-to-iXEN dataset, a cluster of spontaneously differentiating cells were removed from the starting ES cell samples since they are not relevant to the reprogramming in our study.

##### Batch correction:

We did not observe any batch effects amongst the replicates of the XEN-to-iPS dataset and all conditions and replicates from XEN-to-iPS dataset were pooled for downstream analysis. On the other hand, we did observe batch effects in ES-to-iXEN timepoints. Batch effect correction was performed using mnnCorrect^104^ using replicate 1 as the reference. The pooled datasets were analyzed using the same procedure described above with one difference – 2500 highly variable genes were used to account for greater heterogeneity of the data across timepoints. The pooled ES-to-iXEN results demonstrate that batch effects were corrected effectively.

#### Trajectory analysis

Palantir was used for analysis of the XEN-to-iPS and ES-to-iXEN conversion trajectories using default parameters^45^. Palantir models differentiation as a Markov chain and computes for each cell the probability of differentiating to each of the terminal states of the system. The terminal states are also determined automatically by Palantir. Terminal states were specified for the *in vivo* trajectories since end points were known. Palantir first computes diffusion maps, a low dimensional representation of the phenotypic space occupied by the cells. A nearest neighbor graph is then constructed in the diffusion map space. Shortest path distances through this graph from a pre-designated start cell is used to determine a pseudotime ordering of cells. Pseudotime order is then used to transform the nearest neighbor graph into a Markov chain based on which the branch probabilities are computed. The branch probabilities for each cell are summarized using entropy to compute the differentiation potential, a predicted measure of plasticity of the cells.

##### Diffusion maps and MAGIC imputation.

To compute diffusion maps^119–121^ A k-nearest neighbor graph (*k* = 50) was constructed using Euclidean distance with principal components as inputs. The distance matrix representing this graph was converted to an affinity matrix using the adaptive anisotropic kernel i.e., for each cell, distance to *l*^th^ neighbor (*l* (17) < *k* (50)) was used as the scaling factor to account for differences in densities in data. The affinity matrix was normalized to generate the diffusion operator. The top Eigenvectors from the Eigenvalue decomposition of this operator, termed diffusion components, represent the low-dimensional embedding of the data. The number of components was chosen by the Eigen gap among the top Eigen vectors. The same diffusion operator was used for MAGIC^105^ imputation of gene expression data (for t = 3 steps). Single-cell gene expression plots throughout the manuscript use MAGIC imputed expression. Diffusion component computation and MAGIC imputation were performed using the Palantir package (https://github.com/dpeerlab/Palantir).

##### Application of Palantir to characterize transdifferentiation trajectories.

XEN-to-iPS and ES-to-iXEN scRNA-seq data were analyzed separately. A random cell from timepoint 0 was used as the input start cell, which adjusts the start to the nearest extreme of the diffusion components. Palantir automatically determined the terminal states in each trajectory, including a single terminal state in the ES-to-iXEN trajectory, representing the final iXEN state. By contrast, three states – T1, T2 and T3 (final iPS state) – were identified in the XEN-to-iPS trajectory.

##### Comparing XEN-to-iPS reprograming terminal states by differential gene expression analysis.

We sought to identify the genes that are enriched in each of the terminal (and start) states of the XEN-to-iPS trajectory (i.e., Start, T1, T2, T3). For this, we identified the XEN clusters that contained these terminal points (Cluster 2 – XEN/Start, Cluster 4 – T3/iPS, Cluster 7 – T1 and Cluster 11 – T2) and computed the genes that are differentially expressed in each of these clusters compared to rest of the cells in the trajectory using MAST^53^. To summarize the results, we collected the top 50 genes that are significantly differentially expressed (FDR adjusted p-value < 0.01 and logFoldChange > 2) in each of these clusters. We then took the union of all the obtained list of genes and displayed the average z-scored expression of these genes in each of the terminal points as a heatmap using clustermap function in the Seaborn package (Figure S2F).

#### Comparison of the two reprogramming trajectories

To enable a direct comparison between the XEN-to-iPS and ES-to-iXEN transitions, we co-embedded the two trajectories using Harmony (https://github.com/dpeerlab/Harmony)^6^. Harmony augments the nearest neighbor graph within each trajectory (*k* = 50) with mutual nearest neighbors (*k* = 50) between XEN-to-iPS and ES-to-iXEN conversions, and uses them to estimate a joint affinity matrix. The nearest neighbor distance matrix is converted into an affinity matrix using an adaptive Gaussian kernel, where for each cell the affinity is defined as the negative exponential of the distance to a neighbor, scaled by the distance to it’s *l*^th^ neighbor (*l* = *k*/3 = 17). The augmented affinity matrix was used as an input to the Force Directed Layout embedding as implemented in the Harmony package.

The augmented affinity matrix also served as input for computation of diffusion maps using Palantir with default parameters. These diffusion components contain information about both reprogramming trajectories and thus enable a direct comparison between the two. For comparison, we utilized the XEN-to-iPS pseudotime ordering to bin cells along this trajectory into equal sized pseudotime bins. We repeated the same procedure on the ES-to-iXEN trajectory to obtain bins for both trajectories. For each pair of XEN-to-iPS, ES-to-iXEN bins, we computed pairwise distances between each pair of cells using multi-scale distance^45^ applied to the diffusion components. The mean of these distances across all pairs of cells in each bin pair is depicted in Figure S4A.

##### Comparison of in vitro trajectories during intermediate transition phase.

Based on the embedding on the FDL (Figure 4A), we reasoned that while the terminal points of the XEN-to-iPS and ES-to-iXEN trajectories are phenotypically similar, the cells at the transition stages appear distinct. We therefore sought to investigate if XEN-to-iPS and ES-to-iXEN reprogramming follow the same phenotype trajectories. For this, we assumed a batch effect as a source of this discrepancy between the two trajectories and began by aligning them using a completely different algorithm (published at the same time and more widely used to correct batch effects), also called Harmony (default parameters as implemented in Scanpy.external package in Python)^52^. We then performed PhenoGraph clustering as implemented in Scanpy.external package in Python (clustering_algo = leiden, *k* = 30, resolution_parameter = 3) to obtain 29 clusters. From the obtained clusters, we identified those consisting of cells in the transition state (Figure S4B). Among these selected clusters, we computed significantly differential genes between cells transitioning from XEN-to-iPS and ES-to-iXEN using MAST with default parameters^53^, followed by GSEA analysis using GO annotations (http://www.gsea-msigdb.org/gsea/msigdb/human/genesets.jsp?collection=C5).

To ensure our inference is not impacted by the choice of batch correction method, we used an alternative clustering strategy. In particular, we used Harmony^6^ and grouped cells using Spectral Clustering (K-means with n_clusters = 30 as implemented in sklearn package in Python) on the computed diffusion components (top 14 eigenvectors of the joint Markov matrix identified based on the eigengap). We repeated the computation of differentially expressed genes and enriched gene sets and obtained highly similar results. Considering that similar results were obtained using two substantially different computational approaches strengthens the inference that these represent *bone fide* biological differences. We note that the key difference between the Harmony algorithm used throughout the majority of the study^6^ and the batch correction Harmony^52^ is that the former assumes a considerable degree of variation is driven by true biological differences (as would be expected between *in vivo* and *in vitro* data, or cells growing for a different number of days and conditions), whereas the latter often over-corrects and removes true biological differences treating them as batch effects.

#### *Comparison of* in vivo *and* in vitro *trajectories*

The goal of this analysis is to map the path taken by cells in the *in vitro* reprogramming trajectories onto the *in vivo* developmental trajectories to assess whether the *in vivo* developmental states are recapitulated in the *in vitro* trajectories.

##### Construction of the in vivo trajectory.

scRNA-seq data from mouse embryos at the following stages were used for constructing the *in vivo* trajectory: E3.5, E4.5, E5.5, E7.5, E8.75 using ICM, epiblast, primitive & visceral endoderm and parietal endoderm cells^6^. Since the data was generated at discrete timepoints, we used our Harmony algorithm^6^ to connect successive timepoints. Harmony augments affinity matrix derived from the nearest neighbor graph with mutually nearest neighbors between successive timepoints. An affinity matrix is derived from this augmented nearest neighbor graph which serves as input for downstream trajectory analysis and imputation. In addition to using mutually nearest neighbors between successive timepoints, we also utilized mutually nearest neighbors between E4.5 and E7.5 parietal endoderm cells since parietal endoderm cells were not captured from E5.5 and E6.5 in the *in vivo* dataset, and between E7.5 and E8.75 parietal endoderm cells. The augmented affinity matrix was used as input to compute diffusion components and Palantir was used to compute a pseudotime ordering using ICM as the start and epiblast, parietal and visceral endoderm cell states as the terminal state inputs. More specifically, Harmony was applied on the joint PCA embedding (>85% of variance explained) and *n_neighbors = 30*, and Force Directed Layout using *sc.tl.draw_graph* function in Scanpy was run to visualize the augmented embedding.

##### Mapping between in vivo and in vitro trajectories:

Comparison between *in vivo* and *in vitro* trajectories were undertaken separately for XEN-to-iPS and ES-to-iXEN datasets. Following the construction of the *in vitro* trajectory using Palantir and *in vivo* trajectory using Harmony and Palantir, we mapped the two to compare the path taken by *in vitro* cells. First a cell-by-cell affinity matrix was derived using the nearest neighbor graph constructed using all *in vivo* and *in vitro* cells. This affinity matrix was augmented with mutually nearest neighbors between *in vivo* timepoints to recapitulate the *in vivo* trajectory using Harmony^6^. Harmony^6^ was again used to then add mutually neighboring edges between *in vivo* and *in vitro* cells. Thus, the final augmented affinity matrix comprised of the following set of edges: (i) nearest neighbor edges across all cells, (ii) edges between successive *in vivo* timepoints and (iii) edges between *in vivo* and *in vitro* cells. Harmony was applied using default parameters. Highly variable genes (1500) from the *in vivo* trajectory were used for this analysis.

The augmented affinity matrix served as input to compute diffusion maps using Palantir with default parameters. These diffusion components contain information about the *in vivo* and *in vitro* trajectories and the relationship between them, making the comparison feasible. Rather than compare individual cells, we binned the two trajectories into equal pseudotime bins using their respective pseudotime order (Figure S3). For the *in vivo* trajectory, bins were further separated based on cell lineage such as ICM, EPI and others. Cells were binned using 20 intervals. Each *in vitro* bin was mapped to its closest *in vivo* bin using the mean multi-scale distance^45^ between each pair of *in vivo* and *in vitro* cells in the augmented *in vivo*-*in vitro* diffusion space. The median position of the cells in the nearest *in vivo* bin was used for representing *in vitro* bins in Figure 4F and the mean distance is shown in Figure S4E-F.

#### Clustering of gene expression trends

XEN-to-iPS cells were first clustered using PhenoGraph^70^ (*k* = 30) using the principal components as inputs. Differentially expressed genes were identified in each cluster using MAST^53^ with p-value < 1e-5 and log fold change > 1.5. For each cluster, cells from all other clusters were used as a baseline for comparison. Gene expression trends along XEN-to-iPS pseudotime order for each differentially expressed gene were computed using the gene trend analysis as implemented within the Palantir package, which in turn utilizes the Generalized Additive Models (GAMs; gam package in R)^122^. These gene expression trends were then z-scored and used to cluster the genes. This will lead to identification of groups of genes with similar expression dynamics and thus likely represent coordinated gene expression programs. More specifically, we first computed a gene-gene nearest neighbor graph using NearestNeighbor function in sklearn package in Python using “radius = 0.025” and “metric = ‘correlation’” parameters. The distance matrix was then symmetrized and converted into an affinity matrix defined as 1-distance. We then ran Louvain clustering algorithm on the obtained affinity matrix. Finally, we excluded any clusters with fewer than 2 genes to obtain a final set of clusters of genes with similar gene trends.

#### Bulk ATAC-seq analysis

Paired-end sequenced reads from replicate ES and XEN cells were aligned to mouse genome (mm10) with Bowtie2 (version 2.3.4.1)^106^ and “--local –very-sensitive-local -I 10 X 2000” option active. Alignment was followed by filtering of low quality reads (MAPQ<20), duplicate reads, chrM reads and blacklisted regions with the use of Samtools^95^, “MarkDuplicates” from picard tools and bedtools^107^. All filtered reads were corrected for Tn5 insertion at each read end by shifting +4/−5 bp from the positive and negative strand, respectively, and peak calling was performed with MACS2 (version 2.1.1)^108^ ‘—narrow’ option active and default settings. Non-overlapping peaks from replicates were filtered out and only common peaks were used. Peak center (summit file) generated with MACS2 with ‘—narrow’ option was extended to 100bp (+/−50bp) for motif search and all overlapping summits were merged to form an accessibility atlas which was used as background for motif and ChIP enrichment with LOLA R package (see below for LOLA enrichment analysis). For measuring relative accessibility of genes enriched at XEN-to-iPS terminal states, signal from all accessible regions around the promoter regions of selected genes (scRNA-seq gene lists/groups) were summed and compared with the use of Mann-Whitney U test.

#### Single-cell ATAC-seq data processing

##### Data processing and metacell analysis.

Cell Ranger ATAC^109^ was used to preprocess the scATAC-seq data based on GRCm38/mm10 mouse genome, to obtain the sequence alignment files and fragment files, which are then provided as input to the ArchR software^110^. Cell Ranger ATAC performs barcode location detection, sequencing error correction, read alignment, and duplicate read pair identification. The resulting fragment file contains the genomic position information of each sequenced scATAC-seq fragment and the identity of the corresponding cell. With the preprocessed scATAC-seq data in each of the two conversion trajectories (XEN-to-iPS and ES-to-iXEN), ArchR^110^ was used to identify chromatin accessibility peak loci from the data. Each chromatin accessibility peak locus corresponds to an accessible genomic region. Specifically, ArchR uses the input files to generate a sparse count matrix where each row corresponds to a single cell and each column corresponds to a genomic bin (500 base pair). The values in the matrix are the number of fragments in each genomic bin of each cell. ArchR employs the iterative Latent Semantic Indexing (LSI) approach^109,123^ to perform normalization of the sparse count matrix using the frequency-inverse document frequency (TF-IDF) method^124^. We used 100K as the number of variable features for the LSI implementation with the other parameters as default. Next, singular value decomposition (SVD) is applied to the normalized count matrix for dimension reduction of the scATAC-seq data. We used 30 as the number of dimensions after reduction. ArchR then performs clustering of the cells using the graph clustering approach from Seurat^125^ and generates pseudo-bulk replicates based on the cell groups identified from the clustering. More specifically, the data of a set of single cells sharing similarity are merged to create a pseudo-sample to address the sparsity problem of scATAC-seq data. Peak calling was performed using MACS2^108^ on the pseudo-bulk replicates and the iterative overlap peak merging procedure^126^ was applied in ArchR to generate a merged set of chromatin accessibility peak loci for the single cells. Each peak locus is 501bp in length.

The XEN-to-iPS trajectory consists of five timepoints: start, day 7, day 14, day 28, and end (sorted iPS cells), with one replicate for each timepoint. The ES-to-iXEN trajectory consists of five timepoints: start, 9h (h: hour), 24h, 48h, and end (iXEN cells), with two replicates for 24h and 48h and one replicate for each of the other timepoints. The replicates were merged for chromatin accessibility peak loci identification in each trajectory. There are 61040 and 95396 single cells in the scATAC-seq data of the XEN-to-iPS and ES-to-iXEN trajectories, respectively. 257618 and 250671 chromatin accessibility peak loci were identified in either trajectory using ArchR, respectively.

Next, the SEACells algorithm^68^ was used to identify metacells, each of which are representative of a small assembly of single cells sharing the same or similar cell states based on the scATAC-seq data in each conversion trajectory. We identified 543 metacells in the XEN-to-iPS trajectory and 567 metacells in the ES-to-iXEN trajectory (default parameters, except *n_waypoint_eigs = 10*, *waypoint_proportion = 1*). Each metacell is associated with 112 single cells on average or 168 single cells on average in the XEN-to-iPS and ES-to-iXEN trajectories, respectively. The scATAC-seq read counts in each peak locus across the single cells represented by the same metacell were aggregated to approximate the accessibility of the locus in the corresponding metacell, to overcome the limitation of sparsity in scATAC-seq data. The chromatin accessibility count matrix of the scATAC-seq metacells were then normalized – the read count in each peak locus in each metacell was divided by the total read count in the metacell and multiplied by the median of the total counts per metacell across metacells. We performed log transformation of the normalized count matrix with a pseudo-count of 1. The subsequent scATAC-seq data analyses were conducted at the metacell level.

Next, we employed the highly variable gene identification function in the Scanpy package^103^ to detect the peak loci with highly variable accessibility (denoted as highly variable peak loci) from the scATAC-seq data of the metacells. Specifically, dispersion of the chromatin accessibility across the metacells was calculated for each peak locus and normalized within each group of peak loci sharing similar mean accessibility across the metacells. The peak loci with normalized accessibility dispersion and mean accessibility larger than the specified thresholds were selected as a set of highly variable peak loci and were used for representation of the chromatin accessibilities in the metacells in either trajectory, in order to capture more distinctive features across different cell states. We chose a threshold of 3.0 for the normalized accessibility dispersion and a threshold of 0.0125 for mean accessibility in both conversion trajectories. We identified 3584 and 1955 highly variable peak loci with the specified thresholds in the XEN-to-iPS and ES-to-iXEN trajectories, respectively.

For both conversion trajectories, we performed principal component analysis (PCA)^127^ for dimension reduction of normalized and log-transformed chromatin accessibility matrix of highly variable peak loci for the metacells and selected the first 100 principal components (PCs) for feature representation. The force-directed layout (FDL) plots of metacells were generated using the Palantir package^45^ with default parameters for visualization. For the XEN-to-iPS trajectory, PhenoGraph clustering^70^ was applied to the metacells using default parameters (*k* = 30) and the feature representation from highly variable peak loci, identifying 10 clusters (Figure 6B). We further assigned the clusters of metacells to 7 major groups based on the timepoints from which they were collected, and their relative accessibility patterns along pseudotime from the XEN state to the iPS cell state. Specifically, group 1 mostly comprises metacells from the start timepoint. Group 2A and 2B correspond to the two clusters identified for metacells from day 7. Group 3 and 4 (4A and 4B) each contain a mixture of the metacells from day 14 and day 28. The members in group 4B are relatively closer to the iPS cell state while group 4A are more dispersed in the cell states. Group 5 predominantly contains metacells in the iPS cell state. In the ES-to-iXEN trajectory, the cell groups are distinguishable by the associated timepoints, (Figure 6D), and metacells were thus clustered by timepoint.

#### TF binding activity estimation from scATAC-seq data

We used the chromVAR method^69^ to estimate transcription factor (TF) binding activities in each metacell. For each metacell and each TF with binding motifs, chromVAR aggregates the accessibility of the peak loci where the binding motif of the TF is identified, and computes a bias-corrected z-score of the aggregated accessibility as the TF binding activity score in the metacell (noted as chromVAR score). Specifically, the original z-score measures the deviation of the aggregated accessibility for the TF in a metacell from the mean value of the aggregated accessibility across the metacells. For each peak locus containing the TF binding motif, peak loci with GC content and mean chromatin accessibility across the metacells both matching the corresponding locus were sampled genome-wide, forming sets of background peak loci to estimate the background distribution of the z-score for bias-correction. To identify TF binding motifs in the peak loci, we used the curated CIS-BP mouse TF binding motif collection retrieved from the chromVAR repository^69^ and the matchMotifs function in the motifmatchr package (Bioconductor) to perform motif scanning in the sequences of the peak loci, using the threshold of p-value < 5e-5. For both lineage conversion trajectories, we used the chromatin accessibility peak loci with normalized accessibility dispersion greater than 0.5 based on the scATAC-seq data to calculate chromVAR scores for the TFs. We projected chromVAR scores to the force-directed layout of the metacells based on the feature representation from the selected highly variable peak loci as described previously for visualization (Figure 6B and Figure S6A). Higher chromVAR scores correspond to cells with greater relative accessibility of the genomic regions with potential binding sites of the TF queried.

#### Comparison of chromatin accessibility of peak loci across cell groups

We performed differential analysis of chromatin accessibility of selected highly variable peak loci for metacells in the XEN-to-iPS trajectory, to identify peak loci with distinctive accessibility patterns in specific cell groups, or dynamic accessibility patterns across cell groups during the conversion from XEN to iPS cells. There are 7 cell groups (group 1, 2A, 2B, 3, 4A, 4B, and 5) annotated in the XEN-to-iPS trajectory as previously described. We performed two-sample Kolmogorov-Smirnov (K-S) test for each highly variable peak locus to compare the distributions of locus accessibility between a given pair of cell groups, using a threshold of p-value < 1e-4 to identify peak loci that exhibit significant difference in accessibility distributions between the corresponding two groups. More specifically, in Figure 6C, we show the chromatin accessibility changes across the different groups of metacells using the identified differential accessible peak loci between group 4B and group 5 of the metacells in the XEN-to-iPS trajectory.

We further identified chromatin accessibility peak loci that were detected in both XEN-to-iPS and ES-to-iXEN trajectories. Given two peak loci from each of the two trajectories, we define them as existing in both trajectories if they overlap with each other. To increase the number of identified peak loci that overlap between the two trajectories, in addition to the originally selected highly variable peak loci, we also use a threshold of normalized accessibility dispersion above 2.0 (*min_disp* parameter in *pp.highly_variable_genes* in the Scanpy package for detection of highly variable peaks as described above) to select an increased set of highly variable peak loci for both trajectories. We then performed differential accessibility analysis for this set of co-existing peak loci between specific pairs of cell groups in a given trajectory. The chromatin accessibilities of the identified set of shared peak loci across cell groups in the ES-to-iXEN trajectory were visualized by heatmap (Figure 6D). Heatmaps were made using the heatmap.2 function in R.

#### Generating pseudo-bulk ATAC-seq data based on cell groups in the conversion trajectories

Pseudo-bulk ATAC-seq data from the scATAC-seq data were generated based on annotated cell groups in the XEN-to-iPS and ES-to-iXEN trajectories. Each cell group was used to construct a pseudo-bulk sample. In each trajectory, for each cell group and each chromatin accessibility peak locus, we used an average of the chromatin accessibilities of a locus across metacells in the cell group as the accessibility of the locus in the pseudo-bulk ATAC-seq data of the corresponding cell group.

#### ChIP-seq analysis

v6.5 ES and IM8A-1 XEN cells were collected in duplicates at ~25 million each. Cells were crosslinked in 1% PFA in PBS for 10 mins at room temperature and quenched with 125mM glycine for 5 mins at room temperature. Cells were then washed twice with PBS and resuspended in lysis buffer (10mM Tris pH8, 1mM EDTA and 0.5% SDS) at 2×10^7^ cells per 400μl. To shear chromatin, samples were sonicated using a Bioruptor^®^ Pico sonication device (Diagenode) for 12 cycles, 30 seconds on/30 seconds off then pelleted at the maximum speed for 10 mins at 4°C. The supernatant was diluted 5x with dilution buffer (0.01% SDS, 1.1% Triton X-100, 1.2mM EDTA, 16.7mM Tris pH8 and 167mM NaCl), then incubated with primary antibodies at 4°C overnight. Protein G Dynabeads (Invitrogen) were blocked at 4°C overnight using 100 ng per 10μl of beads. The next day, beads were added to samples at 20 μl per sample for 3 hrs at 4°C. Using a magnet to stabilize the beads, they were washed twice in low-salt buffer (0.1% SDS, 1% Triton X-100, 2mM EDTA, 150mM NaCl and 20mM Tris pH8), twice in high-salt buffer (0.1% SDS, 1% Triton X-100, 2mM EDTA, 500mM NaCl and 20mM Tris pH8), twice in LiCl buffer (0.25M LiCl, 1% NP-40, 1% deoxycholic acid, 1mM EDTA and 10mM Tris pH8) and once in TE buffer (10mM Tris pH 8, 0.1mM EDTA). Subsequently, the DNA was eluted from the beads by incubating with 150μl elution buffer (100mM NaHCO_3_ and 1% SDS) for 20 mins at 65°C with vortexing using Eppendorf ThermoMixer C (Eppendorf). The supernatant was collected, reverse crosslinked by incubation overnight at 65°C in the presence of proteinase K (Roche), and cleaned by RNase A (Thermo Scientific) treatment for 1 hr at 37°C; the DNA was purified using a DNA clean and concentrate kit (Zymo Research).

Single-end sequenced reads from replicate ES and XEN cells with corresponding input samples were aligned with the use of Bowtie2 (version 2.3.4.1) to mouse genome (mm10)^106^ using “— local-very-sensitive-local” option. Filtering of low quality reads (MAPQ<20), duplicate reads, chrM reads and blacklisted regions was performed with the use of Samtools^95^, “MarkDuplicates” from picard tools and bedtools^107^. Filtered reads were used to call both ‘narrow’ and ‘broad’ peaks with MACS2 (version 2.1.1)^108^ and default settings with the use of corresponding input for each cell line. Peaks within a distance of a nucleosome were merged into one peak (distance <147 bp) for each replicate. Common peaks between replicates were considered valid and the rest non overlapping peaks were removed from downstream analysis.

#### LOLA enrichment analysis

LOLA (version 1.8.0)^71^ software in R was used to calculate enrichment of transcription factors and histone modifications in ATAC-seq peaks on mouse genome (mm10). LOLA database was expanded based on available published ChIP-seq data for ES and XEN cells. Enrichment of ChIP-seq experiments was estimated by comparing the enrichment of selected accessible regions (late open, gradual, late close, transient) to an atlas of accessible regions generated by merging ES and XEN ATAC-seq peaks from our experiments. Significant enrichment of transcription factors and histone modifications was scored based on p-value levels (<10^−3^).

#### CUT&Run analysis

CUT&RUN on GATA4 and GATA6 was performed on 500,000 3F2 and 3F6 XEN cells in two replicates as previously described with minor modifications^128^. Briefly, BioMag Plus Concanavalin A beads (ConA beads, PolySciences) were washed twice in 100μl cold Bead Activation Buffer (20mM HEPES pH7.5, 10mM KCl, 1mM CaCl_2_, 1mM MnCl_2_). 20μg of ConA beads per CUT&RUN reaction were used. Cells were washed twice in Wash Buffer (20mM HEPES pH7.5, 150mM NaCl, 0.5mM Spermidine (Acros), 1⁄2 protease inhibitor tablet) and bound to activated ConA beads in Wash Buffer for 10 minutes at room temperature. Samples were incubated in PCR strip tubes with 1:50 dilutions of primary antibodies against Gata6 (R&D Systems), Gata4 (Santa Cruz Biotech), or anti-IgG (EpiCypher) in Antibody Buffer (Wash Buffer with 2mM EDTA and 0.01% Digitonin (Millipore Sigma)) overnight with gentle rocking at 4°C. Samples were washed twice in Digitonin Buffer (Washer Buffer with 0.01% Digitonin) and ProteinA/G Mnase (EpiCypher) was bound to samples for 1 hour at 4°C. ProteinA/G Mnase was diluted at 20x in 50μl Antibody Buffer according to manufacturer’s instructions. Samples were washed twice in Digitonin Buffer again and Mnase digestion was activated with 1μl 100mM CaCl_2_ in 50μl Digitonin Buffer for 2 hours. To extract bound chromatin fragments digestion was quenched using 33μl STOP Buffer (340mM NaCl, 20mM EDTA, 4mM EGTA, 50μg/ml RNAse A, 50μg/ml glycogen, 0.015 ng/μl E. coli spike-in) and samples incubated at 37°C for 20 minutes. Chromatin was separated from ConA beads by centrifuging 5 minutes at 16,000g and saving supernatant. DNA was purified by adding 0.1% SDS and 5μg Proteinase K for 10 min at 70°C, performing phenol-chloroform extraction, and precipitation in 100% ethanol at −80°C overnight. Pellets were then washed in 100% ethanol and resuspended in 12μl nuclease free water. CUT&RUN libraries were prepared using the Thruplex Library Prep kit (Takara) according to the manufacturer’s instructions. The number of cycles for library PCR amplification was determined by running 10% of each library sample in an Applied Biosystems qPCR machine with 0.25μl EvaGreen dye (Biotium) for 40 cycles and adding cycles to each sample as described^129^. Libraries were size selected with 1.5x volume SPRIselect beads (Beckman Coulter) and sequenced at the Weill Cornell Genomics Resources Core Facility on an Illumina NovaSeq 6000 (PE-50, 30 million reads per sample).

Paired-end reads were aligned to mouse genome (mm10 version) with bowtie2 (version 2.3.4.1)^106^ and ‘–local –very-sensitive-local’ option active. Picard tools, Samtools^95^ and Bedtools^107^ were used for filtering duplicate reads, low quality reads (MAPQ<20), chrM and blacklisted regions and converting files into sam, bam, bed and bedGraph format. All filtered reads were used to generate bedGraphs and SEACR (version 1.3)^101^ was used to generate peaks with ‘0.01 non stringent’ options active. Peaks not present in all replicates were discarded from downstream analysis.

#### DNA methylation data integration

All aligned filtered reads from the published dataset for ES and XEN cells were annotated on ~17k CpG islands on the mouse genome and signal strength (or enrichment) was calculated for each CpG island. For each ATAC peak cluster, we calculated the CpG island signal strength present within 1.5kb from each ATAC peak and calculated the sum of the CpG strength.

### QUANTIFICATION AND STATISTICAL ANALYSIS

For flow cytometry analysis, FlowJo was used for gating and quantifying populations. Raw image data quantifications were carried out on Imaris (Bitplane) using the Spot model to identify nuclei, and measuring relative fluorescent intensities for each channel. Flow cytometry, image quantification and RT-qPCR data were plotted using R (http://www.r-project.org/).

Details for data processing, quantification and statistical analyses for bulk RNA-seq, bulk ATAC-seq, single-cell RNA-seq, single-cell ATAC-seq, ChIP-set and CUT&Run datasets can be found in the Method Details section. Briefly, differentially expressed genes for bulk RNA-seq data were calculated using the DESeq^97^ or DESeq2^98^ R packages. Differentially expressed genes for single-cell RNA-seq data were computed using MAST^53^. MACS2 (version 2.1.1)^108^ was used for peak calling for bulk ATAC-seq, single-cell ATAC-seq pseudo-bulk replicates, and ChIP-seq datasets. For single-cell ATAC-seq data, ArchR software^110^ was used to identify chromatin accessibility peak loci. LOLA (version 1.8.0) software^71^ was used to calculate enrichment of transcription factor and histone modifications in ATAC-seq peaks on mouse genome (mm10). Relative accessibility of genes enriched at the XEN-to-iPS terminal states was compared using the Mann-Whitney U test. The SEACells algorithm^68^ was used to identify metacells. chromVAR^69^ was used to estimate transcription factor binding activities in metacells.

Where indicated, precision measures and ‘N’ values can be found in the figures or associated figure legends, or in the Results and Methods Details sections.

## Supplementary Material

1

2 **Table S1.** List of differentially expressed genes in terminal states during XEN-to-iPS reprogramming, Related to Figure 3.

3 **Table S2.** List of GO annotations for differentially expressed genes between intermediate states of XEN-to-iPS and ES-to-iXEN conversion trajectories, Related to Figure 4.

4 **Table S3.** List of expression trend clusters of genes along XEN-to-iPS conversion pseudotime, and differentially expressed genes between empty vector and sgGata4 transfected XEN cells, Related to Figure 5.

5 **Table S4.** Highly variable peak loci in scATAC-seq datasets of XEN-to-iPS and ES-to-iXEN conversions, Related to Figure 6.

6 **Table S5.** Peak loci identified as ‘late open’, ‘gradual open’, ‘late close’, and ‘transient open’ from scATAC-seq data fo XEN-to-iPS reprogramming, Related to Figure 6.

7 **Table S6.** List of oligonucleotides and cell plating numbers for reprogramming, Related to STAR Methods.

## Figures and Tables

**Table:** KEY RESOURCES TABLE

REAGENT or RESOURCE	SOURCE	IDENTIFIER
Antibodies		
Rat anti-SOX2 (1:200 dilution)	eBioscience	Cat#14–9811-82; RRID: AB_11219471
Rabbit anti-RFP (1:100 dilution)	Rockland Inc.	Cat#600–401-379; RRID: AB_2209751
Rabbit anti-NANOG (1:500 dilution)	Reprocell	Cat#RCAB002P-F; RRID: AB_1962694
Goat anti-BRACHYURY (1:200 dilution)	R&D Systems	Cat#AF2085; RRID: AB_2200235
Mouse anti-CDX2 (1:200 dilution)	BioGenex	Cat#MU-392AUC; RRID: AB_2650531
Rabbit anti-GATA4 (1:100 dilution)	Santa Cruz	Cat#sc-25310; RRID: AB_627667
Goat anti-GATA6 (1:100 dilution)	R&D Systems	Cat# AF1700; RRID: AB_2108901
Mouse anti-OCT4 (1:100 dilution)	Santa Cruz	Cat# Sc-5279; RRID: AB_628051
Rat anti-CD140a (PDGFRa)::PE-Cy7	eBioscience	Cat#25–1401-82; RRID: AB_2573400
Mouse anti-CD15 (SSEA-1)::AF647	BioLegend	Cat#125608; RRID: AB_1089188
Mouse anti-CD15 (SSEA-1)::BV421	BioLegend	Cat#125614; RRID: AB_2562672
CUTANA IgG Negative Control antibody	EpiCypher	Cat#13–0042; RRID: AB_2923178
Bacterial and virus strains		
NEB 5-alpha Competent E. coli (High efficiency)	NEB	Cat#C2987I
MAX Efficiency DH5⟨ competent cells	Thermo Fisher	Cat#18258012
Chemicals, peptides, and recombinant proteins		
Hoechst	Invitrogen	Cat#H3570
4’,6-diamidino-2-phenylindole (DAPI)	Invitrogen	Cat#D1306
7-AAD viability dye	BioLegend	Cat#420404
Gelatin	Millipore Sigma	Cat#G9391
RPMI 1640 medium	Gibco	Cat#11875093
DMEM medium	Gibco	Cat#11995073
Fetal Bovine Serum (FBS)	VWR	Cat#97068–085
L-glutamine	Gibco	Cat#25030164
Sodium pyruvate	Gibco	Cat#11360070
Penicillin-Streptomycin	Gibco	Cat#15140163
2-mercaptoethanol	Gibco	Cat#21985023
Non-essential amino acids (NEAA)	Gibco	Cat#11140050
0.05% Trypsin-EDTA	Gibco	Cat#25300054
0.25% Trypsin-EDTA	Gibco	Cat#25200056
TrypLE Express Enzyme (1x)	Gibco	Cat#12604013
Doxycycline hyclate	MP Biochemicals	Cat#0219895501
Mitomycin-C	Millipore Sigma	Cat#M4287–2MG
Ascorbic acid	Millipore Sigma	Cat#A4403–100MG
CHIR99021	Reprocell	Cat#040004
TRIzol reagent	Invitrogen	Cat#15596026
PowerUP SYBR Green master mix	Applied Biosystems	Cat#A25742
16% Paraformaldehyde solution	Electron Microscopy Sciences	Cat#15710
EvaGreen Dye	Biotium	Cat#31000
Digitonin	Millipore Sigma	Cat#300410
Triton-X 100	Millipore Sigma	Cat#X100–100ML
Bovine Serum Albumin (BSA)	Millipore Sigma	Cat#A9647
Donkey serum	Jackson ImmunoResearch	Cat#017–000-121
Newborn Calf Serum	Gibco	Cat#16010167
Dynabeads MyOne SILANE	Invitrogen	Cat#37002D
Dynabeads Protein G for Immunoprecipitation	Invitrogen	Cat#10003D
BioMag Plus Concanavalin A	Polysciences	Cat#86057–3
SPRIselect beads	Beckman Coulter	Cat#B23317
Proteinase K	Roche	Cat#03508838103
Rnase A	Thermo Scientific	Cat#EN0531
Spermidine	Acros Organics	Cat#AC132740050
CUTANA pAG-Mnase	EpiCypher	Cat#15–1016
Critical commercial assays		
QuantiTect Reverse Transcription Kit	Qiagen	Cat#205311
Lipofectamine 3000	Invitrogen	Cat#L3000001
NEBuilder HiFi DNA Assembly Master Mix	NEB	Cat#E2621S
10x Genomics Chromium Single Cell 3’ Reagent Kit v2 Chemistry	10x Genomics	Cat#PN-120237
10x Genomics Chromium Next GEN Single Cell ATAC Reagent Kit	10x Genomics	Cat#PN-1000175
Agilent High Sensitivity DNA Kit	Agilent	Cat#5067–4626
DNA Clean and Concentrator-5	Zymo Research	Cat#D4004
Qubit RNA HS Assay Kit	Thermo Scientific	Cat#Q32852
Illumina Ribo-Zero Plus rRNA depletion kit	Illumina	Cat#20040526
NEBNext Ultra Directional RNA Library Prep Kit for Illumina	NEB	Cat#E7760
ThruPLEX DNA-Seq kit	Takara Bio	Cat#R400674
Deposited data		
Single-cell RNA-seq data of XEN-to-iPS and ES-to-iXEN conversions	This study	GSE276704
Single-cell ATAC-seq datasets of XEN-to-iPS and ES-to-iXEN conversions	This study	GSE276702
Bulk RNA-seq data of wild-type ES and XEN cells	Murphy et al.^92^	GSE213645
Bulk RNA-seq data of reprogrammable (‘3F’) XEN and XEN-iPS cells	This study	GSE266451
Bulk RNA-seq data of Gata4/6 KO cells	This study	GSE266451
Bulk ATAC-seq data of wild-type ES and XEN cells	Murphy et al.^92^	GSE213645
Bulk ATAC-seq data of Gata4/6 KO cells	This study	GSE266452
H3K27ac ChIP-seq data from wild-type (v6.5) ES cells	Murphy et al.^92^	GSE213645
H3K27ac ChIP-seq data from wild-type (IM8A-1) XEN cells	Murphy et al.^92^	GSE213645
CUT&Run data of Gata4/6 in XEN cells	This study	GSE266453
Experimental models: Cell lines		
R1 ES cells	Nagy et al.^37^	N.A.
v6.5 ES cells	Rideout et al.^93^	N.A.
IM8A-1 XEN cells	Kunath et al.^11^	N.A.
3F2 XEN	This paper	N.A.
3F4 XEN	This paper	N.A.
3F6 XEN	This paper	N.A.
3F9 XEN	This paper	N.A.
3F2 XEN-iPS	This paper	N.A.
3F4 XEN-iPS#1	This paper	N.A.
3F4 XEN-iPS#2	This paper	N.A.
3F6 XEN-iPS	This paper	N.A.
*Col1a1*^*TetO-Gata4-mCherry/+*^;*R26*^*M2rtTA/+*^;*Gata6*^*H2B-Venus/+*^ ES cells	Schröter et al.^24^ & Freyer et al.^44^	N.A.
Experimental models: Organisms/strains		
Mouse: *Col1A1*^*TetO-OKSmCh/TetO-OKSmCh*^; *R26*^*M2rtTA/M2rtTA*^	Jackson Labs	Stock ID:034917
Mouse: *Oct4-GFP*	Jackson Labs	Stock ID:008214
Mouse: CD1	Charles River Laboratory	022
Mouse: C57BL/6	Jackson Labs	Stock ID: 000664
Oligonucleotides		
See Supplemental Table S6	N.A.	N.A.
Recombinant DNA		
pCX-mCherry	This paper	N.A.
pCX-Gata4-E2A-E2-Crimson	This paper	N.A.
PX458	Addgene	Plasmid#48138
PX458-E2-Crimson	This paper	N.A.
Software and algorithms		
Rstudio/R version 4.1.2 (2021–11-01)	Rstudio	http://www.rproject.org/
ZEN 2.3	Carl Zeiss Microsystems	https://www.zeiss.com/microscopy/en/products/software/zeisszen.html
Fiji/ImageJ	Schindelin et al.^94^	https://imagej.nih.gov/ij/
Imaris 9.1.2	Bitplane	https://imaris.oxinst.com/
SAMtools	Li et al.^95^	N.A.
HTSeq	Anders et al.^96^	N.A.
DESeq	Anders & Huber^97^	N.A.
DESeq2	Love et al.^98^	N.A.
STAR aligner	Dobin et al.^99^	N.A.
featureCounts	Liao et al.^100^	N.A.
SEACR	Meers et al.^101^	N.A.
SEQC	Azizi et al.^102^	N.A.
SCANPY	Wolf et al.^103^	N.A.
Palantir	Setty et al.^45^	https://github.com/dpeerlab/Palantir
mnnCorrect	Haghverdi et al.^104^	N.A.
MAGIC	van Dijk et al.^105^	N.A.
MAST	Finak et al.^53^	N.A.
Harmony	Nowotschin et al.^6^	N.A.
Harmony	Korsunsky et al.^52^	N.A.
PhenoGraph	Levine et al.^70^	N.A.
Bowtie2 (version 2.3.4.1)	Langmead & Salzberg^106^	N.A.
BEDTools	Quinlan & Hall^107^	N.A.
MACS2 (version 2.1.1)	Zhang et al.^108^	N.A.
Cell Ranger ATAC	Satpathy et al.^109^	N.A.
ArchR	Granja et al.^110^	N.A.
SEACells	Persad et al.^68^	N.A.
chromVAR	Schep et al.^69^	N.A.
LOLA (version 1.8.0)	Sheffield & Bock^71^	N.A.
Other		
35mm glass bottom dish	MatTek	Cat#P35G-1.1–14-C)
FlowMi cell strainers (4μm)	Millipore Sigma	Cat#BAH136800040 −50EA
0.35μm Nylon mesh strainer	Falcon	Cat#352235
0.2μm SFCA filter	Thermo Scientific	Cat#7232520

## Highlights:

- OCT4, SOX2 and KLF4-mediated XEN-to-iPS conversion is heterogeneous and inefficient
- Dominant extraembryonic endoderm TF network resists XEN-to-iPS reprogramming
- XEN-to-iPS conversion requires extensive chromatin opening and DNA demethylation
- *In vitro* lineage conversions parallel *in vivo* states during lineage specification

## References

[1] Chazaud C, and Yamanaka Y (2016). Lineage specification in the mouse preimplantation embryo. Dev. Camb. Engl 143, 1063–1074. 10.1242/dev.128314. 27048685

[2] Schrode N, Xenopoulos P, Piliszek A, Frankenberg S, Plusa B, and Hadjantonakis A-K (2013). Anatomy of a blastocyst: cell behaviors driving cell fate choice and morphogenesis in the early mouse embryo. Genes. N. Y. N 2000 51, 219–233. 10.1002/dvg.22368. PMC3633705 23349011

[3] Gardner RL, and Rossant J (1979). Investigation of the fate of 4–5 day post-coitum mouse inner cell mass cells by blastocyst injection. J. Embryol. Exp. Morphol 52, 141–152. 521746

[4] Nowotschin S, Hadjantonakis A-K, and Campbell K (2019). The endoderm: a divergent cell lineage with many commonalities. Dev. Camb. Engl 146, dev150920. 10.1242/dev.150920. PMC6589075 31160415

[5] Chan MM, Smith ZD, Grosswendt S, Kretzmer H, Norman TM, Adamson B, Jost M, Quinn JJ, Yang D, Jones MG, (2019). Molecular recording of mammalian embryogenesis. Nature 570, 77–82. 10.1038/s41586-019-1184-5. 31086336 PMC7229772

[6] Nowotschin S, Setty M, Kuo Y-Y, Liu V, Garg V, Sharma R, Simon CS, Saiz N, Gardner R, Boutet SC, (2019). The emergent landscape of the mouse gut endoderm at single-cell resolution. Nature 569, 361–367. 10.1038/s41586-019-1127-1. 30959515 PMC6724221

[7] Xenopoulos P, Kang M, Puliafito A, Di Talia S, and Hadjantonakis A-K (2015). Heterogeneities in Nanog Expression Drive Stable Commitment to Pluripotency in the Mouse Blastocyst. Cell Rep. 10, 1508–1520. 10.1016/j.celrep.2015.02.010. 25753417 PMC4560681

[8] Kwon GS, Viotti M, and Hadjantonakis A-K (2008). The endoderm of the mouse embryo arises by dynamic widespread intercalation of embryonic and extraembryonic lineages. Dev. Cell 15, 509–520. 10.1016/j.devcel.2008.07.017. 18854136 PMC2677989

[9] Evans MJ, and Kaufman MH (1981). Establishment in culture of pluripotential cells from mouse embryos. Nature 292, 154–156. 10.1038/292154a0. 7242681

[10] Martin G (1981). Isolation of a pluripotent cell line from early mouse embryos cultured in medium conditioned by teratocarcinoma stem cells. Proc. Natl. Acad … 78, 7634–7638. 10.1073/pnas.78.12.7634 PMC349323 6950406

[11] Kunath T, Arnaud D, Uy GD, Okamoto I, Chureau C, Yamanaka Y, Heard E, Gardner RL, Avner P, and Rossant J (2005). Imprinted X-inactivation in extraembryonic endoderm cell lines from mouse blastocysts. Dev. Camb. Engl 132, 1649–1661. 10.1242/dev.01715. 15753215

[12] Garg V, Morgani S, and Hadjantonakis A-K (2016). Capturing Identity and Fate Ex Vivo: Stem Cells from the Mouse Blastocyst. Curr. Top. Dev. Biol 120, 361–400. 10.1016/bs.ctdb.2016.04.007. 27475857

[13] Watts J, Lokken A, Moauro A, and Ralston A (2018). Capturing and Interconverting Embryonic Cell Fates in a Dish. Curr. Top. Dev. Biol 128, 181–202. 10.1016/bs.ctdb.2017.11.008. 29477163 PMC7092685

[14] Amadei G, Handford CE, Qiu C, De Jonghe J, Greenfeld H, Tran M, Martin BK, Chen D-Y, Aguilera-Castrejon A, Hanna JH, (2022). Embryo model completes gastrulation to neurulation and organogenesis. Nature 610, 143–153. 10.1038/s41586-022-05246-3. 36007540 PMC9534772

[15] Sozen B, Amadei G, Cox A, Wang R, Na E, Czukiewska S, Chappell L, Voet T, Michel G, Jing N, (2018). Self-assembly of embryonic and two extra-embryonic stem cell types into gastrulating embryo-like structures. Nat. Cell Biol 20, 979–989. 10.1038/s41556-018-0147-7. 30038254

[16] Tarazi S, Aguilera-Castrejon A, Joubran C, Ghanem N, Ashouokhi S, Roncato F, Wildschutz E, Haddad M, Oldak B, Gomez-Cesar E, (2022). Post-gastrulation synthetic embryos generated ex utero from mouse naive ESCs. Cell 185, 3290–3306.e25. 10.1016/j.cell.2022.07.028. 35988542 PMC9439721

[17] Weatherbee BAT, Gantner CW, Iwamoto-Stohl LK, Daza RM, Hamazaki N, Shendure J, and Zernicka-Goetz M (2023). Pluripotent stem cell-derived model of the post-implantation human embryo. Nature 622, 584–593. 10.1038/s41586-023-06368-y. 37369347 PMC10584688

[18] Zhang S, Chen T, Chen N, Gao D, Shi B, Kong S, West RC, Yuan Y, Zhi M, Wei Q, (2019). Implantation initiation of self-assembled embryo-like structures generated using three types of mouse blastocyst-derived stem cells. Nat. Commun 10, 496. 10.1038/s41467-019-08378-9. 30700702 PMC6353907

[19] Zheng Y, Xue X, Shao Y, Wang S, Esfahani SN, Li Z, Muncie JM, Lakins JN, Weaver VM, Gumucio DL, (2019). Controlled modelling of human epiblast and amnion development using stem cells. Nature 573, 421–425. 10.1038/s41586-019-1535-2. 31511693 PMC8106232

[20] Buganim Y, and Jaenisch R (2012). Transdifferentiation by defined factors as a powerful research tool to address basic biological questions. Cell Cycle Georget. Tex 11, 4485–4486. 10.4161/cc.22665. PMC3562283 23165203

[21] Xu J, Du Y, and Deng H (2015). Direct lineage reprogramming: strategies, mechanisms, and applications. Cell Stem Cell 16, 119–134. 10.1016/j.stem.2015.01.013. 25658369

[22] Fujikura J, Yamato E, Yonemura S, Hosoda K, Masui S, Nakao K, Miyazaki Ji J, and Niwa H (2002). Differentiation of embryonic stem cells is induced by GATA factors. Genes Dev. 16, 784–789. 10.1101/gad.968802. 11937486 PMC186328

[23] Shimosato D, Shiki M, and Niwa H (2007). Extra-embryonic endoderm cells derived from ES cells induced by GATA factors acquire the character of XEN cells. BMC Dev. Biol 7, 1–12. 10.1186/1471-213X-7-80. 17605826 PMC1933422

[24] Schröter C, Rué P, Mackenzie JP, and Martinez Arias A (2015). FGF/MAPK signaling sets the switching threshold of a bistable circuit controlling cell fate decisions in embryonic stem cells. Dev. Camb. Engl 142, 4205–4216. 10.1242/dev.127530. PMC4689219 26511924

[25] Wamaitha SE, del Valle I, Cho LTY, Wei Y, Fogarty NME, Blakeley P, Sherwood RI, Ji H, and Niakan KK (2015). Gata6 potently initiates reprograming of pluripotent and differentiated cells to extraembryonic endoderm stem cells. Genes Dev. 29, 1239–1255. 10.1101/gad.257071.114. 26109048 PMC4495396

[26] Bessonnard S, De Mot L, Gonze D, Barriol M, Dennis C, Goldbeter A, Dupont G, and Chazaud C (2014). Gata6, Nanog and Erk signaling control cell fate in the inner cell mass through a tristable regulatory network. Dev. Camb. Engl 141, 3637–3648. 10.1242/dev.109678. 25209243

[27] Schrode N, Saiz N, Di Talia S, and Hadjantonakis A-K (2014). GATA6 levels modulate primitive endoderm cell fate choice and timing in the mouse blastocyst. Dev. Cell 29, 454–467. 10.1016/j.devcel.2014.04.011. 24835466 PMC4103658

[28] Zhao Y, Zhao T, Guan J, Zhang X, Fu Y, Ye J, Zhu J, Meng G, Ge J, Yang S, (2015). A XEN-like State Bridges Somatic Cells to Pluripotency during Chemical Reprogramming. Cell 163, 1678–1691. 10.1016/j.cell.2015.11.017. 26686652

[29] Zhao T, Fu Y, Zhu J, Liu Y, Zhang Q, Yi Z, Chen S, Jiao Z, Xu X, Xu J, (2018). Single-Cell RNA-Seq Reveals Dynamic Early Embryonic-like Programs during Chemical Reprogramming. Cell Stem Cell, 1–15. 10.1016/j.stem.2018.05.025. 29937202

[30] Guan J, Wang G, Wang J, Zhang Z, Fu Y, Cheng L, Meng G, Lyu Y, Zhu J, Li Y, (2022). Chemical reprogramming of human somatic cells to pluripotent stem cells. Nature 605, 325–331. 10.1038/s41586-022-04593-5. 35418683

[31] Parenti A, Halbisen MA, Wang K, Latham K, and Ralston A (2016). OSKM Induce Extraembryonic Endoderm Stem Cells in Parallel to Induced Pluripotent Stem Cells. Stem Cell Rep. 6, 447–455. 10.1016/j.stemcr.2016.02.003. PMC4834035 26947975

[32] Schiebinger G, Shu J, Tabaka M, Cleary B, Subramanian V, Solomon A, Gould J, Liu S, Lin S, Berube P, (2019). Optimal-Transport Analysis of Single-Cell Gene Expression Identifies Developmental Trajectories in Reprogramming. Cell 176, 928943.e22. 10.1016/j.cell.2019.01.006. PMC6402800 30712874

[33] Bar-Nur O, Brumbaugh J, Verheul C, Apostolou E, Pruteanu-Malinici I, Walsh RM, Ramaswamy S, and Hochedlinger K (2014). Small molecules facilitate rapid and synchronous iPSC generation. Nat. Methods 11, 1170–1176. 10.1038/nmeth.3142. 25262205 PMC4326224

[34] Stadtfeld M, Maherali N, Borkent M, and Hochedlinger K (2010). A reprogrammable mouse strain from gene-targeted embryonic stem cells. Nat. Methods 7, 53–55. 10.1038/nmeth.1409. 20010832 PMC3987893

[35] Beard C, Hochedlinger K, Plath K, Wutz A, and Jaenisch R (2006). Efficient method to generate single-copy transgenic mice by site-specific integration in embryonic stem cells. Genes. N. Y. N 2000 44, 23–28. 10.1002/gene.20180. 16400644

[36] Lengner CJ, Camargo FD, Hochedlinger K, Welstead GG, Zaidi S, Gokhale S, Scholer HR, Tomilin A, and Jaenisch R (2007). Oct4 expression is not required for mouse somatic stem cell self-renewal. Cell Stem Cell 1, 403–415. 10.1016/j.stem.2007.07.020. 18159219 PMC2151746

[37] Nagy A, Rossant J, Nagy R, Abramow-Newerly W, and Roder JC (1993). Derivation of completely cell culture-derived mice from early-passage embryonic stem cells. Proc. Natl. Acad. Sci. U. S. A 90, 8424–8428. 10.1073/pnas.90.18.8424. 8378314 PMC47369

[38] Cui L, Johkura K, Yue F, Ogiwara N, Okouchi Y, Asanuma K, and Sasaki K (2004). Spatial distribution and initial changes of SSEA-1 and other cell adhesion-related molecules on mouse embryonic stem cells before and during differentiation. J. Histochem. Cytochem. Off. J. Histochem. Soc 52, 1447–1457. 10.1369/jhc.3A6241.2004. PMC3957812 15505339

[39] Solter D, and Knowles BB (1978). Monoclonal antibody defining a stage-specific mouse embryonic antigen (SSEA-1). Proc. Natl. Acad. Sci. U. S. A 75, 5565–5569. 10.1073/pnas.75.11.5565. 281705 PMC393007

[40] Artus J, Panthier J-J, and Hadjantonakis A-K (2010). A role for PDGF signaling in expansion of the extra-embryonic endoderm lineage of the mouse blastocyst. Dev. Camb. Engl 137, 3361–3372. 10.1242/dev.050864. PMC2947752 20826533

[41] Plusa B, Piliszek A, Frankenberg S, Artus J, and Hadjantonakis A-K (2008). Distinct sequential cell behaviours direct primitive endoderm formation in the mouse blastocyst. Dev. Camb. Engl 135, 3081–3091. 10.1242/dev.021519. PMC2768606 18725515

[42] Rugg-Gunn PJ, Cox BJ, Lanner F, Sharma P, Ignatchenko V, McDonald ACH, Garner J, Gramolini AO, Rossant J, and Kislinger T (2012). Cell-surface proteomics identifies lineage-specific markers of embryo-derived stem cells. Dev. Cell 22, 887–901. 10.1016/j.devcel.2012.01.005. 22424930 PMC3405530

[43] McDonald ACH, Biechele S, Rossant J, and Stanford WL (2014). Sox17-mediated XEN cell conversion identifies dynamic networks controlling cell-fate decisions in embryo-derived stem cells. Cell Rep. 9, 780–793. 10.1016/j.celrep.2014.09.026. 25373912

[44] Freyer L, Schröter C, Saiz N, Schrode N, Nowotschin S, Martinez-Arias A, and Hadjantonakis A-K (2015). A loss-of-function and H2B-Venus transcriptional reporter allele for Gata6 in mice. BMC Dev. Biol 15, 38. 10.1186/s12861-015-0086-5. 26498761 PMC4619391

[45] Setty M, Kiseliovas V, Levine J, Gayoso A, Mazutis L, and Pe’er D (2019). Characterization of cell fate probabilities in single-cell data with Palantir. Nat. Biotechnol 37, 451–460. 10.1038/s41587-019-0068-4. 30899105 PMC7549125

[46] Liu J, Han Q, Peng T, Peng M, Wei B, Li D, Wang X, Yu S, Yang J, Cao S, (2015). The oncogene c-Jun impedes somatic cell reprogramming. Nat. Cell Biol 17, 856–867. 10.1038/ncb3193. 26098572

[47] Markov GJ, Mai T, Nair S, Shcherbina A, Wang YX, Burns DM, Kundaje A, and Blau HM (2021). AP-1 is a temporally regulated dual gatekeeper of reprogramming to pluripotency. Proc. Natl. Acad. Sci. U. S. A 118. 10.1073/pnas.2104841118. PMC8201948 34088849

[48] Chronis C, Fiziev P, Papp B, Butz S, Bonora G, Sabri S, Ernst J, and Plath K (2017). Cooperative Binding of Transcription Factors Orchestrates Reprogramming. Cell 168, 442–459.e20. 10.1016/j.cell.2016.12.016. 28111071 PMC5302508

[49] Nichols J, Zevnik B, Anastassiadis K, Niwa H, Klewe-Nebenius D, Chambers I, Schöler H, and Smith A (1998). Formation of Pluripotent Stem Cells in the Mammalian Embryo Depends on the POU Transcription Factor Oct4. Cell 95, 379–391. 10.1016/S0092-8674(00)81769-9. 9814708

[50] Rogers MB, Hosler BA, and Gudas LJ (1991). Specific expression of a retinoic acid-regulated, zinc-finger gene, Rex-1, in preimplantation embryos, trophoblast and spermatocytes. Dev. Camb. Engl 113, 815–824. 10.1242/dev.113.3.815. 1821852

[51] Schöler HR, Hatzopoulos AK, Balling R, Suzuki N, and Gruss P (1989). A family of octamer-specific proteins present during mouse embryogenesis: evidence for germline-specific expression of an Oct factor. EMBO J. 8, 2543–2550. 10.1002/j.1460-2075.1989.tb08392.x. 2573523 PMC401252

[52] Korsunsky I, Millard N, Fan J, Slowikowski K, Zhang F, Wei K, Baglaenko Y, Brenner M, Loh P-R, and Raychaudhuri S (2019). Fast, sensitive and accurate integration of single-cell data with Harmony. Nat. Methods 16, 1289–1296. 10.1038/s41592-019-0619-0. 31740819 PMC6884693

[53] Finak G, McDavid A, Yajima M, Deng J, Gersuk V, Shalek AK, Slichter CK, Miller HW, McElrath MJ, Prlic M, (2015). MAST: A flexible statistical framework for assessing transcriptional changes and characterizing heterogeneity in single-cell RNA sequencing data. Genome Biol. 16, 278. 10.1186/s13059-015-0844-5. 26653891 PMC4676162

[54] Senner CE, Krueger F, Oxley D, Andrews S, and Hemberger M (2012). DNA methylation profiles define stem cell identity and reveal a tight embryonic-extraembryonic lineage boundary. Stem Cells Dayt. Ohio 30, 2732–2745. 10.1002/stem.1249. 23034951

[55] Rugg-Gunn PJ, Cox BJ, Ralston A, and Rossant J (2010). Distinct histone modifications in stem cell lines and tissue lineages from the early mouse embryo. Proc. Natl. Acad. Sci. U. S. A 107, 10783–10790. 10.1073/pnas.0914507107. 20479220 PMC2890770

[56] Gatie MI, and Kelly GM (2018). Metabolic profile and differentiation potential of extraembryonic endoderm-like cells. Cell Death Discov. 5, 42. 10.1038/s41420-018-0102-1. PMC6158286 30302276

[57] Gatie MI, Cooper TT, Khazaee R, Lajoie GA, and Kelly GM (2022). Lactate Enhances Mouse ES Cell Differentiation Toward XEN Cells In Vitro. Stem Cells Dayt. Ohio 40, 239–259. 10.1093/stmcls/sxab022. 35323987

[58] Mulvey CM, Schröter C, Gatto L, Dikicioglu D, Fidaner IB, Christoforou A, Deery MJ, Cho LTY, Niakan KK, Martinez-Arias A, (2015). Dynamic Proteomic Profiling of Extra-Embryonic Endoderm Differentiation in Mouse Embryonic Stem Cells. Stem Cells Dayt. Ohio 33, 2712–2725. 10.1002/stem.2067. 26059426

[59] Artus J, Douvaras P, Piliszek A, Isern J, Baron MH, and Hadjantonakis A-K (2012). BMP4 signaling directs primitive endoderm-derived XEN cells to an extraembryonic visceral endoderm identity. Dev. Biol 361, 245–262. 10.1016/j.ydbio.2011.10.015. 22051107 PMC3246571

[60] Brown K, Legros S, Artus J, Doss MX, Khanin R, Hadjantonakis A-K, and Foley A (2010). A comparative analysis of extra-embryonic endoderm cell lines. PloS One 5, e12016. 10.1371/journal.pone.0012016. PMC2919048 20711519

[61] Kruithof-de Julio M, Alvarez MJ, Galli A, Chu J, Price SM, Califano A, and Shen MM (2011). Regulation of extra-embryonic endoderm stem cell differentiation by Nodal and Cripto signaling. Dev. Camb. Engl 138, 3885–3895. 10.1242/dev.065656. PMC3160087 21862554

[62] Anderson KGV, Hamilton WB, Roske FV, Azad A, Knudsen TE, Canham MA, Forrester LM, and Brickman JM (2017). Insulin fine-tunes self-renewal pathways governing naive pluripotency and extra-embryonic endoderm. Nat. Cell Biol 19, 1164–1177. 10.1038/ncb3617. 28945231

[63] Linneberg-Agerholm M, Wong YF, Romero Herrera JA, Monteiro RS, Anderson KGV, and Brickman JM (2019). Naïve human pluripotent stem cells respond to Wnt, Nodal and LIF signalling to produce expandable naïve extra-embryonic endoderm. Dev. Camb. Engl 146. 10.1242/dev.180620. 31740534

[64] Ohinata Y, Endo TA, Sugishita H, Watanabe T, Iizuka Y, Kawamoto Y, Saraya A, Kumon M, Koseki Y, Kondo T, (2022). Establishment of mouse stem cells that can recapitulate the developmental potential of primitive endoderm. Science 375, 574–578. 10.1126/science.aay3325. 35113719

[65] Zhong Y, Choi T, Kim M, Jung KH, Chai YG, and Binas B (2018). Isolation of primitive mouse extraembryonic endoderm (pXEN) stem cell lines. Stem Cell Res. 30, 100–112. 10.1016/j.scr.2018.05.008. 29843002

[66] De Mot L, Gonze D, Bessonnard S, Chazaud C, Goldbeter A, and Dupont G (2016). Cell Fate Specification Based on Tristability in the Inner Cell Mass of Mouse Blastocysts. Biophys. J 110, 710–722. 10.1016/j.bpj.2015.12.020. 26840735 PMC4744165

[67] Saiz N, Mora-Bitria L, Rahman S, George H, Herder JP, Garcia-Ojalvo J, and Hadjantonakis A-K (2020). Growth-factor-mediated coupling between lineage size and cell fate choice underlies robustness of mammalian development. eLife 9, 1042–1044. 10.7554/eLife.56079. PMC7513828 32720894

[68] Persad S, Choo Z-N, Dien C, Sohail N, Masilionis I, Chaligné R, Nawy T, Brown CC, Sharma R, Pe’er I, (2023). SEACells infers transcriptional and epigenomic cellular states from single-cell genomics data. Nat. Biotechnol 41, 1746–1757. 10.1038/s41587-023-01716-9. 36973557 PMC10713451

[69] Schep AN, Wu B, Buenrostro JD, and Greenleaf WJ (2017). chromVAR: inferring transcription-factor-associated accessibility from single-cell epigenomic data. Nat. Methods 14, 975–978. 10.1038/nmeth.4401. 28825706 PMC5623146

[70] Levine JH, Simonds EF, Bendall SC, Davis KL, Amir ED, Tadmor MD, Litvin O, Fienberg HG, Jager A, Zunder ER, (2015). Data-Driven Phenotypic Dissection of AML Reveals Progenitor-like Cells that Correlate with Prognosis. Cell 162, 184–197. 10.1016/j.cell.2015.05.047. 26095251 PMC4508757

[71] Sheffield NC, and Bock C (2016). LOLA: enrichment analysis for genomic region sets and regulatory elements in R and Bioconductor. Bioinforma. Oxf. Engl 32, 587–589. 10.1093/bioinformatics/btv612. PMC4743627 26508757

[72] Eminli S, Foudi A, Stadtfeld M, Maherali N, Ahfeldt T, Mostoslavsky G, Hock H, and Hochedlinger K (2009). Differentiation stage determines potential of hematopoietic cells for reprogramming into induced pluripotent stem cells. Nat. Genet 41, 968–976. 10.1038/ng.428. 19668214 PMC3987895

[73] Tan KY, Eminli S, Hettmer S, Hochedlinger K, and Wagers AJ (2011). Efficient generation of iPS cells from skeletal muscle stem cells. PloS One 6, e26406. 10.1371/journal.pone.0026406. PMC3196574 22028872

[74] Graf T, and Enver T (2009). Forcing cells to change lineages. Nature 462, 587–594. 10.1038/nature08533. 19956253

[75] Vierbuchen T, and Wernig M (2011). Direct lineage conversions: unnatural but useful? Nat. Biotechnol 29, 892–907. 10.1038/nbt.1946. 21997635 PMC3222779

[76] Zhou Q, and Melton DA (2008). Extreme makeover: converting one cell into another. Cell Stem Cell 3, 382–388. 10.1016/j.stem.2008.09.015. 18940730

[77] Li X, Liu D, Ma Y, Du X, Jing J, Wang L, Xie B, Sun D, Sun S, Jin X, (2017). Direct Reprogramming of Fibroblasts via a Chemically Induced XEN-like State. Cell Stem Cell, 1–10. 10.1016/j.stem.2017.05.019. 28648365

[78] Liuyang S, Wang G, Wang Y, He H, Lyu Y, Cheng L, Yang Z, Guan J, Fu Y, Zhu J, (2023). Highly efficient and rapid generation of human pluripotent stem cells by chemical reprogramming. Cell Stem Cell 30, 450–459.e9. 10.1016/j.stem.2023.02.008. 36944335

[79] Guo S, Zi X, Schulz VP, Cheng J, Zhong M, Koochaki SHJ, Megyola CM, Pan X, Heydari K, Weissman SM, (2014). Nonstochastic reprogramming from a privileged somatic cell state. Cell 156, 649–662. 10.1016/j.cell.2014.01.020. 24486105 PMC4318260

[80] Hanna J, Saha K, Pando B, van Zon J, Lengner CJ, Creyghton MP, van Oudenaarden A, and Jaenisch R (2009). Direct cell reprogramming is a stochastic process amenable to acceleration. Nature 462, 595–601. 10.1038/nature08592. 19898493 PMC2789972

[81] Stadtfeld M, Maherali N, Breault DT, and Hochedlinger K (2008). Defining molecular cornerstones during fibroblast to iPS cell reprogramming in mouse. Cell Stem Cell 2, 230–240. 10.1016/j.stem.2008.02.001. 18371448 PMC3538379

[82] Sridharan R, Tchieu J, Mason MJ, Yachechko R, Kuoy E, Horvath S, Zhou Q, and Plath K (2009). Role of the murine reprogramming factors in the induction of pluripotency. Cell 136, 364–377. 10.1016/j.cell.2009.01.001. 19167336 PMC3273494

[83] Polo JM, Anderssen E, Walsh RM, Schwarz B. a., Nefzger CM, Lim SM, Borkent M, Apostolou E, Alaei S, Cloutier J, (2012). A molecular roadmap of reprogramming somatic cells into iPS cells. Cell 151, 1617–1632. 10.1016/j.cell.2012.11.039. 23260147 PMC3608203

[84] Bernstein BE, Mikkelsen TS, Xie X, Kamal M, Huebert DJ, Cuff J, Fry B, Meissner A, Wernig M, Plath K, (2006). A bivalent chromatin structure marks key developmental genes in embryonic stem cells. Cell 125, 315–326. 10.1016/j.cell.2006.02.041. 16630819

[85] Tosenberger A, Gonze D, Bessonnard S, Cohen-Tannoudji M, Chazaud C, and Dupont G (2017). A multiscale model of early cell lineage specification including cell division. NPJ Syst. Biol. Appl 3, 16. 10.1038/s41540-017-0017-0. 28649443 PMC5466652

[86] Artus J, Piliszek A, and Hadjantonakis A-K (2011). The primitive endoderm lineage of the mouse blastocyst: sequential transcription factor activation and regulation of differentiation by Sox17. Dev. Biol 350, 393–404. 10.1016/j.ydbio.2010.12.007. 21146513 PMC3461954

[87] Takagi N, and Sasaki M (1975). Preferential inactivation of the paternally derived X chromosome in the extraembryonic membranes of the mouse. Nature 256, 640–642. 10.1038/256640a0. 1152998

[88] Hudson QJ, Seidl CIM, Kulinski TM, Huang R, Warczok KE, Bittner R, Bartolomei MS, and Barlow DP (2011). Extra-embryonic-specific imprinted expression is restricted to defined lineages in the post-implantation embryo. Dev. Biol 353, 420–431. 10.1016/j.ydbio.2011.02.017. 21354127 PMC3081948

[89] Ilgren EB (1980). Polyploidization of extraembryonic tissues during mouse embryogenesis. J. Embryol. Exp. Morphol 59, 103–111. 7217865

[90] Tarkowski AK, Witkowska A, and Opas J (1977). Development of cytochalasin in B-induced tetraploid and diploid/tetraploid mosaic mouse embryos. J. Embryol. Exp. Morphol 41, 47–64. 10.1242/dev.41.1.47. 591878

[91] Eakin GS, Hadjantonakis A-K, Papaioannou VE, and Behringer RR (2005). Developmental potential and behavior of tetraploid cells in the mouse embryo. Dev. Biol 288, 150–159. 10.1016/j.ydbio.2005.09.028. 16246322

[92] Murphy D, Salataj E, Di Giammartino DC, Rodriguez-Hernaez J, Kloetgen A, Garg V, Char E, Uyehara CM, Ee L-S, Lee U, (2024). 3D Enhancer-promoter networks provide predictive features for gene expression and coregulation in early embryonic lineages. Nat. Struct. Mol. Biol 31, 125–140. 10.1038/s41594-023-01130-4. 38053013 PMC10897904

[93] Rideout WM, Wakayama T, Wutz A, Eggan K, Jackson-Grusby L, Dausman J, Yanagimachi R, and Jaenisch R (2000). Generation of mice from wild-type and targeted ES cells by nuclear cloning. Nat. Genet 24, 109–110. 10.1038/72753. 10655052

[94] Schindelin J, Arganda-Carreras I, Frise E, Kaynig V, Longair M, Pietzsch T, Preibisch S, Rueden C, Saalfeld S, Schmid B, (2012). Fiji: an open-source platform for biological-image analysis. Nat. Methods 9, 676–682. 10.1038/nmeth.2019. 22743772 PMC3855844

[95] Li H, Handsaker B, Wysoker A, Fennell T, Ruan J, Homer N, Marth G, Abecasis G, Durbin R, and 1000 Genome Project Data Processing Subgroup (2009). The Sequence Alignment/Map format and SAMtools. Bioinforma. Oxf. Engl 25, 2078–2079. 10.1093/bioinformatics/btp352. PMC2723002 19505943

[96] Anders S, Pyl PT, and Huber W (2015). HTSeq--a Python framework to work with high-throughput sequencing data. Bioinforma. Oxf. Engl 31, 166–169. 10.1093/bioinformatics/btu638. PMC4287950 25260700

[97] Anders S, and Huber W (2010). Differential expression analysis for sequence count data. Genome Biol. 11, R106. 10.1186/gb-2010-11-10-r106. 20979621 PMC3218662

[98] Love MI, Huber W, and Anders S (2014). Moderated estimation of fold change and dispersion for RNA-seq data with DESeq2. Genome Biol. 15. 10.1186/s13059-014-0550-8. PMC4302049 25516281

[99] Dobin A, Davis CA, Schlesinger F, Drenkow J, Zaleski C, Jha S, Batut P, Chaisson M, and Gingeras TR (2013). STAR: Ultrafast universal RNA-seq aligner. Bioinformatics 29, 15–21. 10.1093/bioinformatics/bts635. 23104886 PMC3530905

[100] Liao Y, Smyth GK, and Shi W (2014). featureCounts: an efficient general purpose program for assigning sequence reads to genomic features. Bioinforma. Oxf. Engl 30, 923–930. 10.1093/bioinformatics/btt656. 24227677

[101] Meers MP, Tenenbaum D, and Henikoff S (2019). Peak calling by Sparse Enrichment Analysis for CUT&RUN chromatin profiling. Epigenetics Chromatin 12, 42. 10.1186/s13072-019-0287-4. 31300027 PMC6624997

[102] Azizi E, Carr AJ, Plitas G, Cornish AE, Konopacki C, Prabhakaran S, Nainys J, Wu K, Kiseliovas V, Setty M, (2018). Single-Cell Map of Diverse Immune Phenotypes in the Breast Tumor Microenvironment. Cell 174, 1293–1308.e36. 10.1016/j.cell.2018.05.060. 29961579 PMC6348010

[103] Wolf FA, Angerer P, and Theis FJ (2018). SCANPY: Large-scale single-cell gene expression data analysis. Genome Biol. 19, 15. 10.1186/s13059-017-1382-0. 29409532 PMC5802054

[104] Haghverdi L, Lun ATL, Morgan MD, and Marioni JC (2018). Batch effects in single-cell RNA-sequencing data are corrected by matching mutual nearest neighbors. Nat. Biotechnol 36, 421–427. 10.1038/nbt.4091. 29608177 PMC6152897

[105] van Dijk D, Sharma R, Nainys J, Yim K, Kathail P, Carr AJ, Burdziak C, Moon KR, Chaffer CL, Pattabiraman D, (2018). Recovering Gene Interactions from Single-Cell Data Using Data Diffusion. Cell 174, 716–729.e27. 10.1016/j.cell.2018.05.061. 29961576 PMC6771278

[106] Langmead B, and Salzberg SL (2012). Fast gapped-read alignment with Bowtie 2. Nat. Methods 9, 357–359. 10.1038/nmeth.1923. 22388286 PMC3322381

[107] Quinlan AR, and Hall IM (2010). BEDTools: a flexible suite of utilities for comparing genomic features. Bioinforma. Oxf. Engl 26, 841–842. 10.1093/bioinformatics/btq033. PMC2832824 20110278

[108] Zhang Y, Liu T, Meyer CA, Eeckhoute J, Johnson DS, Bernstein BE, Nusbaum C, Myers RM, Brown M, Li W, (2008). Model-based analysis of ChIP-Seq (MACS). Genome Biol. 9, R137. 10.1186/gb-2008-9-9-r137. 18798982 PMC2592715

[109] Satpathy AT, Granja JM, Yost KE, Qi Y, Meschi F, McDermott GP, Olsen BN, Mumbach MR, Pierce SE, Corces MR, (2019). Massively parallel single-cell chromatin landscapes of human immune cell development and intratumoral T cell exhaustion. Nat. Biotechnol 37, 925–936. 10.1038/s41587-019-0206-z. 31375813 PMC7299161

[110] Granja JM, Corces MR, Pierce SE, Bagdatli ST, Choudhry H, Chang HY, and Greenleaf WJ (2021). ArchR is a scalable software package for integrative single-cell chromatin accessibility analysis. Nat. Genet 53, 935. 10.1038/s41588-021-00850-x. PMC8187146 33790476

[111] Niakan KK, Schrode N, Cho LTY, and Hadjantonakis A-K (2013). Derivation of extraembryonic endoderm stem (XEN) cells from mouse embryos and embryonic stem cells. Nat. Protoc 8, 1028–1041. 10.1038/nprot.2013.049. 23640167 PMC3927835

[112] Czechanski A, Byers C, Greenstein I, Schrode N, Donahue LR, Hadjantonakis AK, and Reinholdt LG (2014). Derivation and characterization of mouse embryonic stem cells from permissive and nonpermissive strains. Nat. Protoc 9, 559–574. 10.1038/nprot.2014.030. 24504480 PMC4112089

[113] Niwa H, Yamamura K, and Miyazaki J (1991). Efficient selection for high-expression transfectants with a novel eukaryotic vector. Gene 108, 193–199. 10.1016/0378-1119(91)90434-D. 1660837

[114] Nowotschin S, Eakin GS, and Hadjantonakis A-K (2009). Dual transgene strategy for live visualization of chromatin and plasma membrane dynamics in murine embryonic stem cells and embryonic tissues. Genes. N. Y. N 2000 47, 330–336. 10.1002/dvg.20500. PMC2875877 19358158

[115] Okabe M, Ikawa M, Kominami K, Nakanishi T, and Nishimune Y (1997). “Green mice” as a source of ubiquitous green cells. FEBS Lett. 407, 313–319. 10.1016/S0014-5793(97)00313-X. 9175875

[116] Ran FA, Hsu PD, Wright J, Agarwala V, Scott DA, and Zhang F (2013). Genome engineering using the CRISPR-Cas9 system. Nat. Protoc 8, 2281–2308. 10.1038/nprot.2013.143. 24157548 PMC3969860

[117] Labun K, Montague TG, Krause M, Torres Cleuren YN, Tjeldnes H, and Valen E (2019). CHOPCHOP v3: expanding the CRISPR web toolbox beyond genome editing. Nucleic Acids Res. 47, W171–W174. 10.1093/nar/gkz365. 31106371 PMC6602426

[118] Zheng GXY, Terry JM, Belgrader P, Ryvkin P, Bent ZW, Wilson R, Ziraldo SB, Wheeler TD, McDermott GP, Zhu J, (2017). Massively parallel digital transcriptional profiling of single cells. Nat. Commun 8, 14049. 10.1038/ncomms14049. 28091601 PMC5241818

[119] Haghverdi L, Buettner F, and Theis FJ (2015). Diffusion maps for high-dimensional single-cell analysis of differentiation data. Bioinforma. Oxf. Engl 31, 2989–2998. 10.1093/bioinformatics/btv325. 26002886

[120] Setty M, Tadmor MD, Reich-Zeliger S, Angel O, Salame TM, Kathail P, Choi K, Bendall S, Friedman N, and Pe’er D (2016). Wishbone identifies bifurcating developmental trajectories from single-cell data. Nat. Biotechnol 34, 637–645. 10.1038/nbt.3569. 27136076 PMC4900897

[121] Coifman RR, and Lafon S (2006). Diffusion maps. Appl. Comput. Harmon. Anal 21, 5–30. 10.1016/j.acha.2006.04.006.

[122] Hastie TJ, and Tibshirani RJ (1990). Generalized Additive Models (CRC press). 10.1177/096228029500400302 8548102

[123] Granja JM, Klemm S, McGinnis LM, Kathiria AS, Mezger A, Corces MR, Parks B, Gars E, Liedtke M, Zheng GXY, (2019). Single-cell multiomic analysis identifies regulatory programs in mixed-phenotype acute leukemia. Nat. Biotechnol 37, 1458–1465. 10.1038/s41587-019-0332-7. 31792411 PMC7258684

[124] Cusanovich DA, Daza R, Adey A, Pliner HA, Christiansen L, Gunderson KL, Steemers FJ, Trapnell C, and Shendure J (2015). Multiplex single cell profiling of chromatin accessibility by combinatorial cellular indexing. Science 348, 910–914. 10.1126/science.aab1601. 25953818 PMC4836442

[125] Hao Y, Hao S, Andersen-Nissen E, Mauck WM, Zheng S, Butler A, Lee MJ, Wilk AJ, Darby C, Zager M, (2021). Integrated analysis of multimodal single-cell data. Cell 184, 3573–3587.e29. 10.1016/j.cell.2021.04.048. 34062119 PMC8238499

[126] Corces MR, Trevino AE, Hamilton EG, Greenside PG, Sinnott-Armstrong NA, Vesuna S, Satpathy AT, Rubin AJ, Montine KS, Wu B, (2017). An improved ATAC-seq protocol reduces background and enables interrogation of frozen tissues. Nat. Methods 14, 959–962. 10.1038/nmeth.4396. 28846090 PMC5623106

[127] Jolliffe IT, and Cadima J (2016). Principal component analysis: a review and recent developments. Philos. Transact. A Math. Phys. Eng. Sci 374, 20150202. 10.1098/rsta.2015.0202. PMC4792409 26953178

[128] Skene PJ, Henikoff JG, and Henikoff S (2018). Targeted in situ genome-wide profiling with high efficiency for low cell numbers. Nat. Protoc 13, 1006–1019. 10.1038/nprot.2018.015. 29651053

[129] Buenrostro JD, Wu B, Chang HY, and Greenleaf WJ (2015). ATAC-seq: A Method for Assaying Chromatin Accessibility Genome-Wide. Curr. Protoc. Mol. Biol 109, 21.29.1–21.29.9. 10.1002/0471142727.mb2129s109. PMC4374986 25559105

