# Supplementary material for: Single-cell analysis of bidirectional reprogramming between early embryonic states identify mechanisms of differential lineage plasticities in mice
Source: Dev Cell. Author manuscript; Available in PMC 2025 Apr 15. (PMC11998022; doi:10.1016/j.devcel.2024.11.022)
Supplement: 1 [file NIHMS2043813-supplement-1.pdf]

Supplemental Information

Single-cell analysis of bidirectional reprogramming between early embryonic states identify mechanisms of differential lineage plasticities in mice

Vidur Garg, Yang Yang, Sonja Nowotschin, Manu Setty, Eralda Salataj, Ying-Yi Kuo, Dylan Murphy, Roshan Sharma, Amy Jang, Alexander Polyzos, Dana Pe'er,
Effie Apostolou, Anna-Katerina Hadjantonakis

Garg et al., **Supplementary Figure 1**

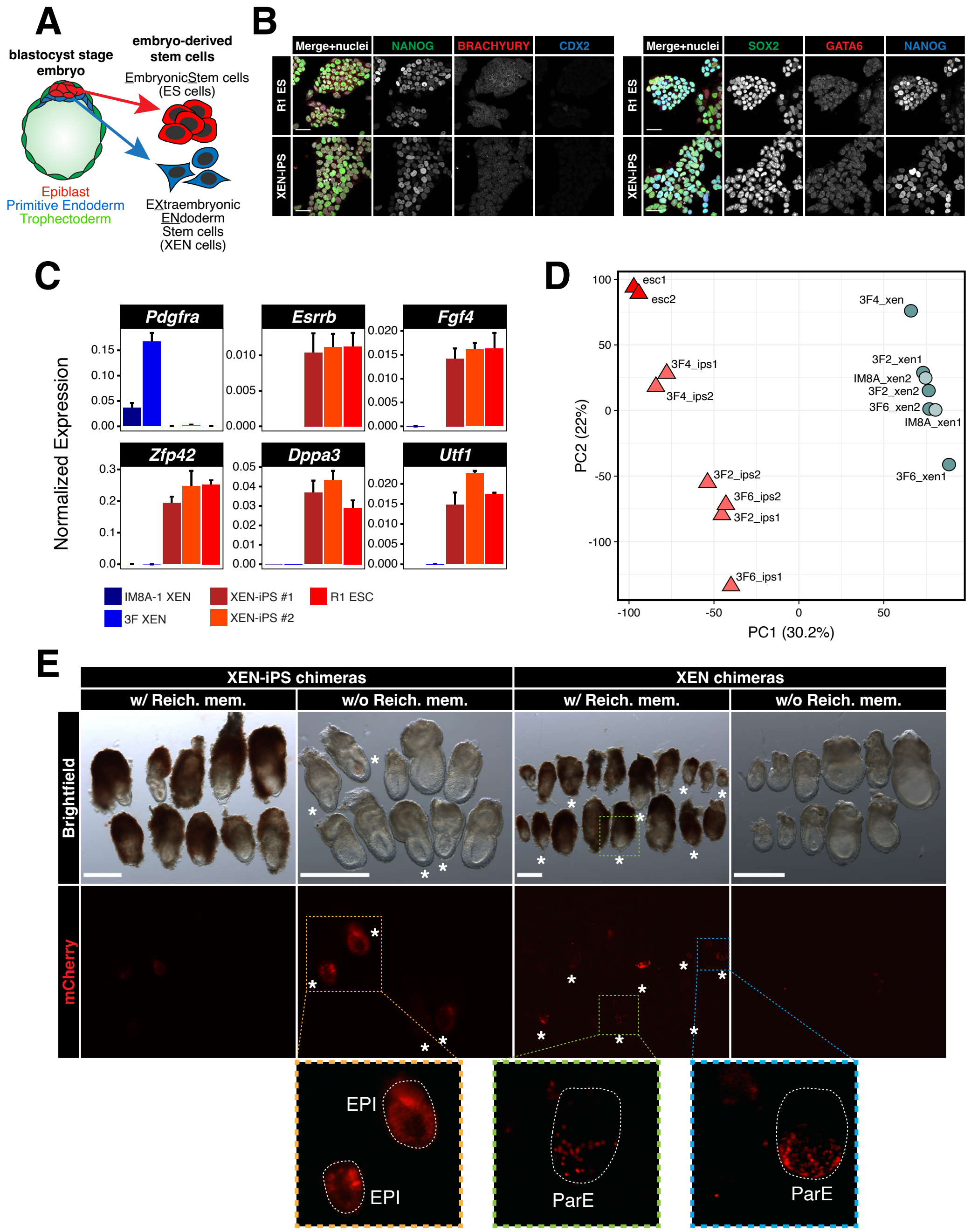

Figure S1. XEN-iPS cells display comparable characteristics and differentiation potential to wild-type ES cells, Related to Figure 1.

(A) Schematic illustrating the embryo lineage of origin for embryonic stem (ES) cells and extraembryonic endoderm stem (XEN) cells.

(B) Immunofluorescence staining of wild-type ES and XEN-iPS cells with markers of naïve pluripotency, primitive streak, mesoderm and endoderm. Scale bars represent 50μm.

(C) RT-qPCR gene expression data for several XEN and ES cell markers in wild-type XEN (IM8A-1), 3F XEN, two XEN-iPS lines (#1 and #2), and wild-type ES cells (R1). Individual bars show mean expression of three technical replicates normalized to mean expression of two reference genes: *Actb* and *Gapdh*; error bars represent standard deviation.

(D) Principle component analysis (PCA) of bulk RNA-seq data from XEN-iPS, wild-type ES, and XEN cells. Data was projected onto the top two PCs explaining 30.2% and 22% of the variance, respectively.

(E) Wholemount brightfield and epifluorescence images of XEN-iPS chimeric embryos with and without Reichardt's membrane demonstrating lack of contribution of XEN-iPS cells to parietal endoderm (ParE)- and visceral endoderm (VE)-derived lineages *(left)*, and specific contribution of XEN cells to the ParE lineages *(right)*. Scale bars represent 500μm.

Garg et al., Supplementary Figure 2

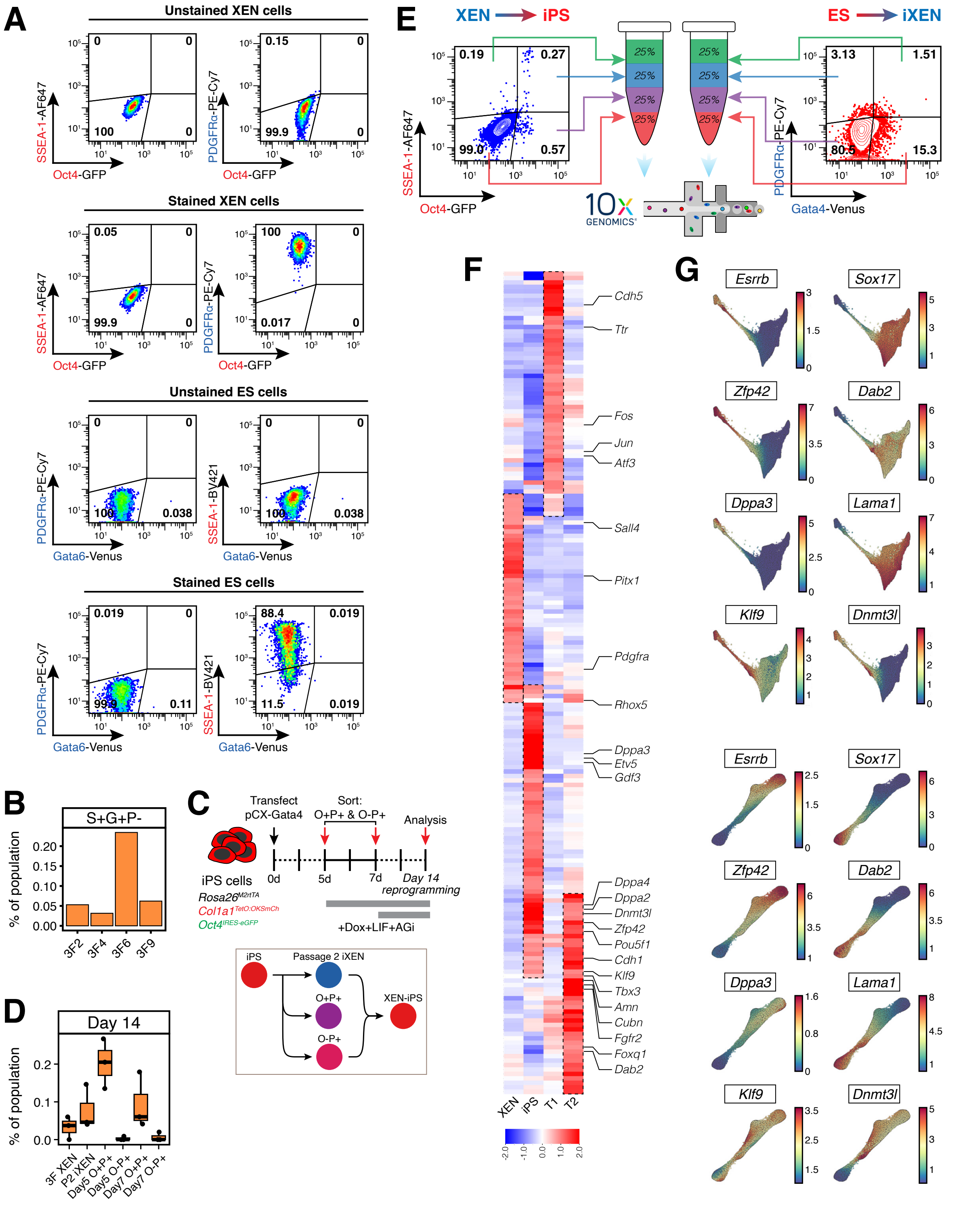

Figure S2. Reciprocal lineage conversions of XEN and ES cells have drastically different kinetics and efficiencies of conversion, Related to Figures 2 & 3.

(A) Gating logic to assess marker expression in live, singlet cells/events. Unstained versus stained XEN or ES cells were used to determine positive and negative gates for each marker.

(B) Bar chart showing reprogramming efficiency of four different 3F XEN lines. Efficiency was calculated as percentage of total reprogramming cells that are SSEA-1+/*Oct4*-GFP+/PDGFRα- following 28 days of reprogramming.

(C) Tracking reprogramming potential of O+P+ and O-P+ subpopulations sorted at days 5 and 7 following transfection with a Gata4-E2-Crimson expression vector for ES/iPS-to-iXEN conversion. Sorted cells and passage 2 (i.e. nascent) iXEN cells were re-plated for reprogramming for 14 days; percentage of iPS-like cells was determined using flow cytometry in (D).

(D) Box plots showing proportion of the entire population that were S+O+P- at day 14 of reprogramming. Middle line marks the median; lower and upper hinges mark first and third quartiles; whiskers extend to 1.5*interquartile range (IQR) from the hinge. N = 3.

(E) Fluorescence-activated cell sorting strategy used prior to encapsulation of Day 7, Day 14 and Day 28 samples of XEN-to-iPS conversion, and 9h sample of ES-to-iXEN conversion. Indicated cell populations were reconstituted to ~25% each in final pool of cells assayed for scRNA-seq.

(F) Heatmap of pseudo-bulk differential gene expression between XEN, XEN-iPS, T1 and T2 states. Scale indicates average z-score.

(G) Gene expression of XEN and pluripotency-associated markers. Each cell is colored by its MAGIC imputed expression level.

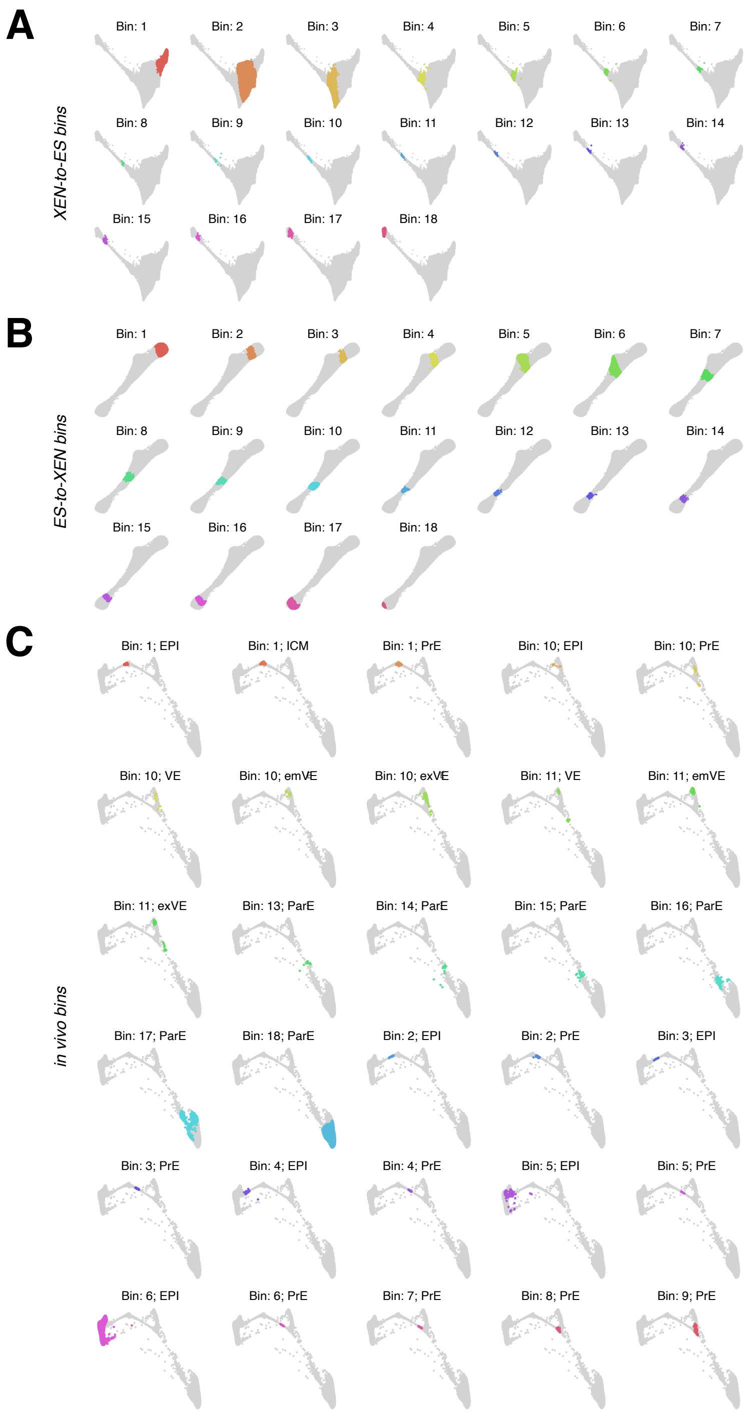
A
XEN-to-ES bins
Bin: 1
Bin: 2
Bin: 3
Bin: 4
Bin: 5
Bin: 6
Bin: 7
Bin: 8
Bin: 9
Bin: 10
Bin: 11
Bin: 12
Bin: 13
Bin: 14
Bin: 15
Bin: 16
Bin: 17
Bin: 18
B
ES-to-XEN bins
Bin: 1
Bin: 2
Bin: 3
Bin: 4
Bin: 5
Bin: 6
Bin: 7
Bin: 8
Bin: 9
Bin: 10
Bin: 11
Bin: 12
Bin: 13
Bin: 14
Bin: 15
Bin: 16
Bin: 17
Bin: 18
C
in vivo bins
Bin: 1; EPI
Bin: 1; ICM
Bin: 1; PrE
Bin: 10; EPI
Bin: 10; PrE
Bin: 10; VE
Bin: 10; emVE
Bin: 10; exVE
Bin: 11; VE
Bin: 11; emVE
Bin: 11; exVE
Bin: 13; ParE
Bin: 14; ParE
Bin: 15; ParE
Bin: 16; ParE
Bin: 17; ParE
Bin: 18; ParE
Bin: 2; EPI
Bin: 2; PrE
Bin: 3; EPI
Bin: 3; PrE
Bin: 4; EPI
Bin: 4; PrE
Bin: 5; EPI
Bin: 5; PrE
Bin: 6; EPI
Bin: 6; PrE
Bin: 7; PrE
Bin: 8; PrE
Bin: 9; PrE

Figure S3. XEN-to-iPS and ES-to-iXEN conversions follow similar trajectories that approximate *in vivo* cell states, Related to Figure 4.

(A) Force-directed layout of the XEN-to-iPS trajectory, with individual bins used for trajectory comparisons.

(B) Force-directed layout of the ES-to-iXEN trajectory, with individual bins used for trajectory comparisons.

(C) Force-directed layout of the *in vivo* trajectory, with individual bins used for trajectory comparisons.

Garg et al., Supplementary Figure 4

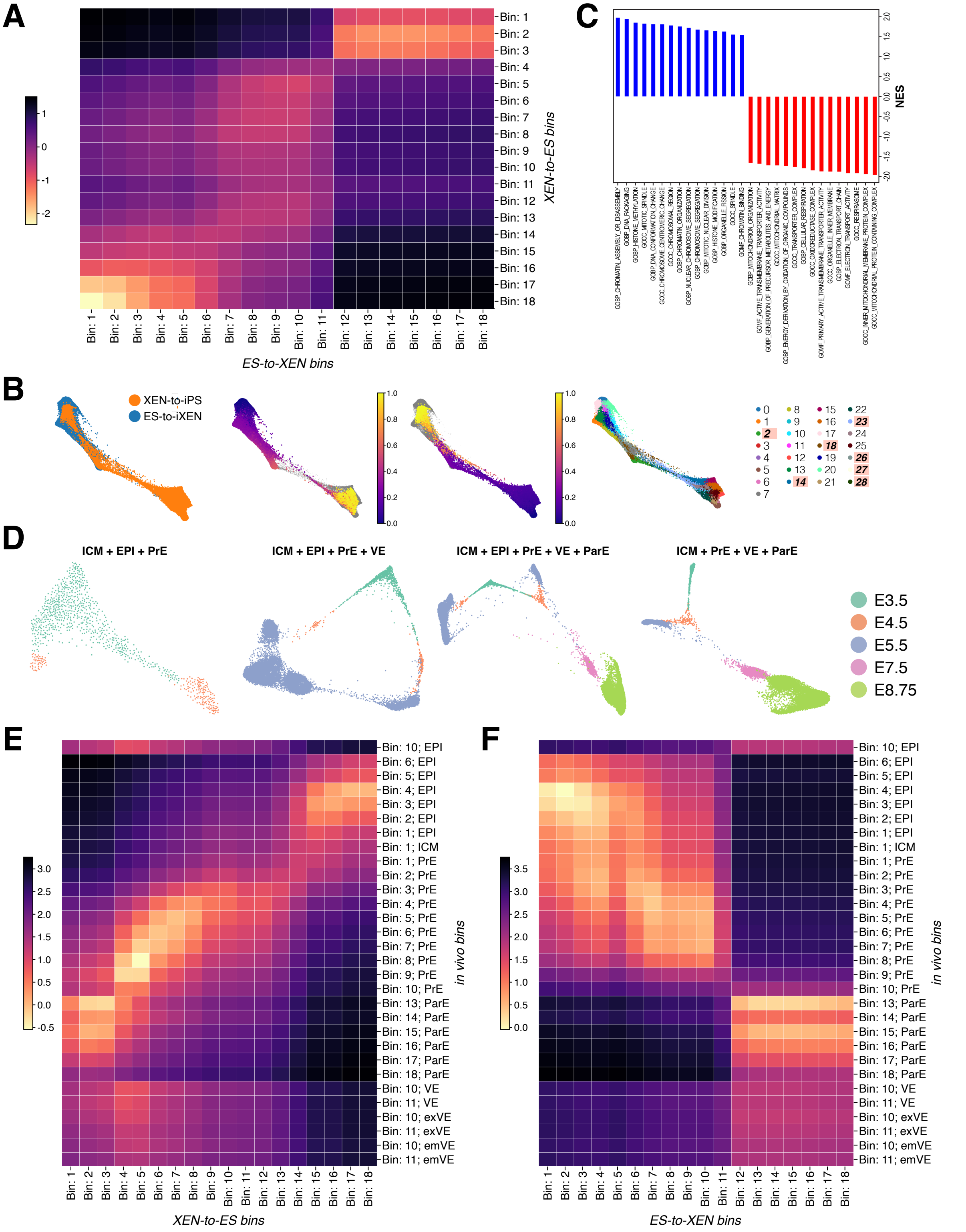

Figure S4. XEN-to-iPS and ES-to-iXEN conversions follow similar trajectories that approximate *in vivo* cell states, Related to Figure 4.

(A) Heatmap of the relative similarity of different XEN-to-iPS bins to ES-to-iXEN bins. Scale represents relative distance in phenotypic space (lower distance corresponds to increased similarity).

(B) Force-directed layout of combined XEN-to-iPS and ES-to-iXEN trajectories based on Harmony integration[1], colored by (from left to right) trajectory, XEN-to-iPS pseudotime, ES-to-iXEN pseudotime, and PhenoGraph clusters. Intermediate state clusters used to calculate differential gene expression in (C) are highlighted.

(C) Normalized enrichment scores (NES) of gene ontology pathways calculated for genes upregulated in the ES-to-iXEN trajectory (blue) among intermediate state clusters in (B), or in the XEN-to-iPS trajectory (red).

(D) Force-directed layouts of combined *in vivo* stages and lineages as labeled above and color-coded as indicated by stage.

(E) Heatmap of the distance between XEN-to-iPS bins to *in vivo* bins. Each entry is the log scaled average diffusion distance between every pair of cells in the two bins being compared.

(F) Heatmap representation of the distance between ES-to-iXEN bins to *in vivo* bins. Each entry is the log scaled average diffusion distance between every pair of cells in the two bins being compared.

Garg et al., Supplementary Figure 5

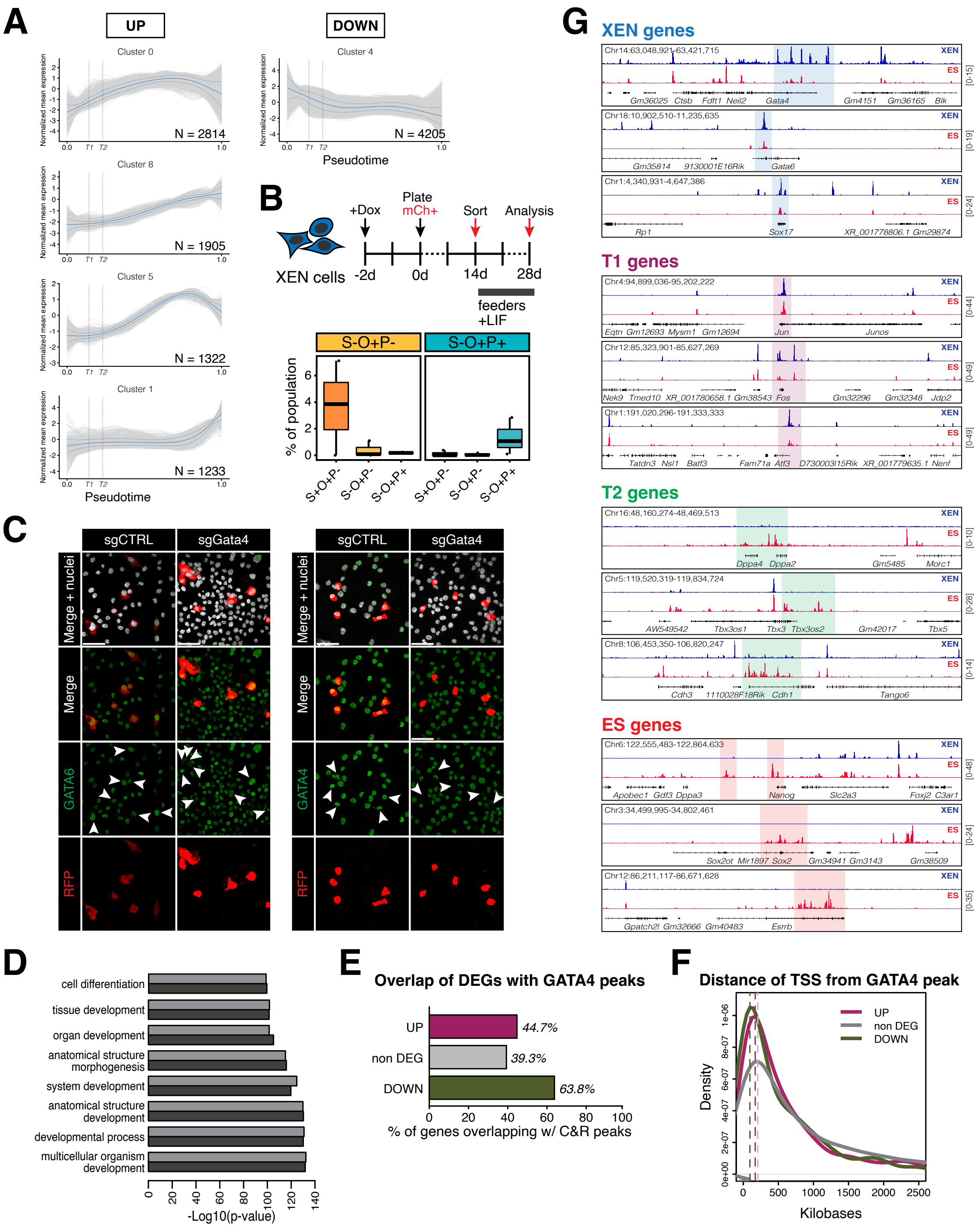

Figure S5. XEN transcriptional network presents a roadblock for XEN-to-iPS reprogramming, Related to Figure 5.

(A) Gene expression waves over pseudotime of XEN-to-iPS reprogramming. Plots show expression trend of individual genes within each cluster (grey lines). Solid blue line represents mean expression of all genes in the respective cluster. Dotted blue lines represent $\pm$ 1 s.d. Vertical dotted lines indicate T1 and T2 terminal states along the pseudotime axis.

(B) *(Top)* Tracking the reprogramming potential of *Oct4*-GFP expressing subpopulations in the absence of continued transgene expression. S-O+P- and S-O+P+ subpopulations sorted on day 14 of reprogramming were re-plated in the absence of doxycycline and AGi. Percentage of iPS-like cells was determined after an additional 14 days using flow cytometry. *(Bottom)* Box plots depicting the proportion of the entire population represented by each displayed subpopulation at day 28. N = 3.

(C) Immunofluorescence staining of XEN cells transfected with Cas9/sgRNA expression vectors targeting *Gata4* or empty vector control. White arrowheads indicate transfected cells counterstained with anti-RFP antibody. Scale bars represent 50μm.

(D) Gene ontology analysis of DEGs following *Gata4* knockout, as shown in Figure 5F. Different shades represent data from 2 different sgRNAs tested.

(E) Bar chart showing the percentage of DEGs overlapping with GATA4 CUT&RUN peaks.

(F) GATA4 peak distribution relative to TSS of DEGs or non DEGs following *Gata4* KO.

(G) Example IGV tracks showing ATAC-seq peaks at specific genomic loci surrounding genes representing XEN, ES, T1 and T2 terminal states. Signal values are indicated to the right.

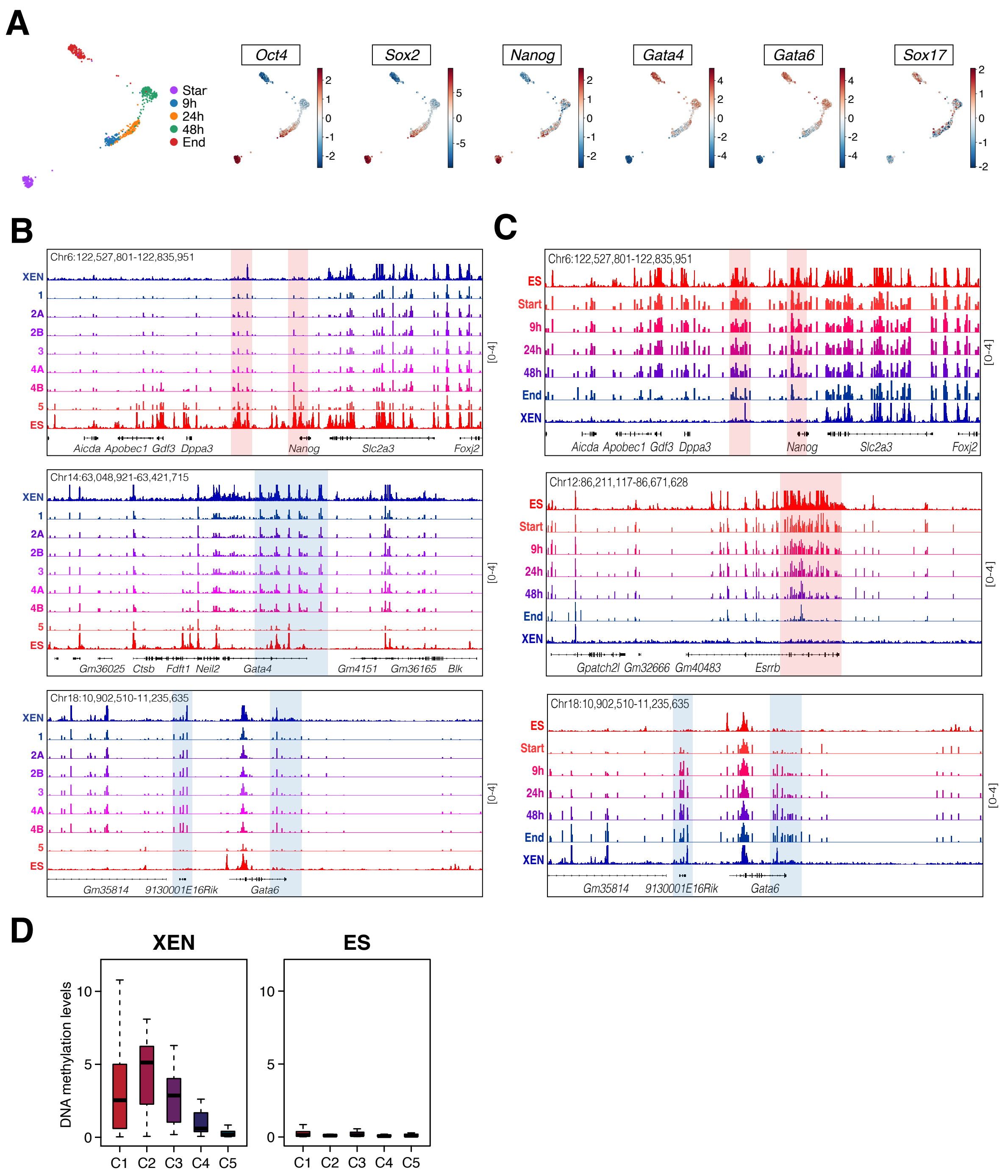
A
Star
9h
24h
48h
End
Oct4
Sox2
Nanog
Gata4
Gata6
Sox17
B
Chr6:122,527,801-122,835,951
XEN
1
2A
2B
3
4A
4B
5
ES
Aicda Apobec1 Gdf3 Dppa3
Nanog
Slc2a3
Foxj2
[0-4]
Chr14:63,048,921-63,421,715
Gm36025 Ctsb Fdft1 Neil2
Gata4
Gm4151 Gm36165 Blk
Chr18:10,902,510-11,235,635
Gm35814 9130001E16Rik
Gata6
C
ES
Start
9h
24h
48h
End
XEN
Chr12:86,211,117-86,671,628
Gpatch2l Gm32666 Gm40483
Esrrb
D
XEN
ES
DNA methylation levels
C1 C2 C3 C4 C5

Figure S6. Establishing an EPI-like chromatin state underlies the inefficient XEN-to-iPS conversion, Related to Figure 6.

(A) Force-directed layouts showing the combined scATAC-seq metacell data for ES-to-iXEN conversion and highlighting the individual timepoints *(left)*, or ChromVAR scores for ES- and XEN-specific TFs *(right)*. Number of metacells = 567.

(B) Example IGV tracks showing accessibility peaks of pseudo-bulk scATAC-seq data of XEN-to-iPS conversion. Highlighted are relative accessibility in metacell groups at specific genomic loci surrounding XEN- and ES-specific genes. Signal values are indicated to the right.

(C) Example IGV tracks showing accessibility peaks of pseudo-bulk scATAC-seq data of ES-to-iXEN conversion. Highlighted are relative accessibility at individual timepoints and at specific genomic loci surrounding ES- and XEN-specific genes. Signal values are indicated to the right.

(D) Boxplots showing DNA methylation levels in XEN and ES cells of the highly variable loci depicted in Figure 6C.

SUPPLEMENTAL TABLES

Table S1. List of differentially expressed genes in terminal states during XEN-to-iPS reprogramming, Related to Figure 3.

Table S2. List of GO annotations for differentially expressed genes between intermediate states of XEN-to-iPS and ES-to-iXEN conversion trajectories, Related to Figure 4.

Table S3. List of expression trend clusters of genes along XEN-to-iPS conversion pseudotime, and differentially expressed genes between empty vector and sgGata4 transfected XEN cells, Related to Figure 5.

Table S4. Highly variable peak loci in scATAC-seq datasets of XEN-to-iPS and ES-to-iXEN conversions, Related to Figure 6.

Table S5. Peak loci identified as 'late open', 'gradual open', 'late close', and 'transient open' from scATAC-seq data fo XEN-to-iPS reprogramming, Related to Figure 6.

Table S6. List of oligonucleotides and cell plating numbers for reprogramming, Related to STAR Methods.

Supplemental References

1. Korsunsky, I., Millard, N., Fan, J., Slowikowski, K., Zhang, F., Wei, K., Baglaenko, Y., Brenner, M., Loh, P.-R., and Raychaudhuri, S. (2019). Fast, sensitive and accurate integration of single-cell data with Harmony. Nat. Methods *16*, 1289–1296. https://doi.org/10.1038/s41592-019-0619-0.
